# Supplementary material for: Identification of an oncogenic network with prognostic and therapeutic value in prostate cancer
Source: Mol Syst Biol. 2018 Aug 14;14(8):e8202. doi: 10.15252/msb.20188202 (PMC6684952; doi:10.15252/msb.20188202)
Supplement: Supplementary file 1 — Appendix [file MSB-14-e8202-s001.pdf]

Appendix

Table of contents

Page	Appendix Figures	Description
2	S1	Meta-analysis of human gene expression profiling arrays identifies gene modules that correlate with prostate cancer progression.
3	S2	shGFP control samples for the gene expression array are not significantly enriched among WGCNA modules.
4	S3	The green module was not enriched for full length AR regulated genes.
5	S4	Expression of AR-V7 generated a slight growth defect on the yeast strain.
6	S5	Network analysis of AR-V7 interactome was obtained using STRING of hits identified by SGA screening.
7	S6	Pathway analysis of the seven gene set reveals a strong association to cell cycle.
8	S7	The seven genes are co-expressed in human PC samples, but do not correlate with full length AR expression levels.
9	S8	Elevated expression of the seven gene set is associated with higher Gleason scores and other adverse indicators in human PC samples.
11	S9	The seven gene set does not have prognostic value in several other types of cancer.
12	S10	None of the seven genes are regulated by ligand-activated full length AR.
14	S11	The seven gene set is not associated to full length AR levels in human samples.
15	S12	Stable depletion of the expression of each of the seven genes reduces CRPC cell proliferation in two different cell lines.
16	S13	Analysis of efficacy of shRNA-mediated depletion of the expression of each of the seven genes.
17	S14	Members of the gene set modify AR-V7 and full length AR expression levels.
18	S15	The compound N-9 affects directly and indirectly four of the seven genes due to pathway interactions.

Appendix Figure S1

A

Dataset Identifier	Array	Tissue	Number of samples	Reference
EMEXP1243	U133plus2	Human	81	Traka et al., 2008
GSE17951	U133plus2	Human	154	Jia et al., 2011
GSE32982	U133plus2	Human	9	Vaarala et al., 2012
GSE3325	U133plus2	Human	19	Varambally et al., 2005
GSE45016	U133plus2	Human	11	Satake et al., 2010
GSE46602	U133plus2	Human	50	Mortensen et al., 2015
GSE55945	U133plus2	Human	21	Arredouani et al., 2009
GSE7307	U133plus2	Human	30	Rands et al 2013.,
		**TOTAL**	**375**	

B

**Phenotype**	**Number of samples**
Normal Prostate Tissue	129
Benign Prostatic Hyperplasia (BPH)	25
High-Grade Prostatic Intraepithelial neoplasia (HGPIN)	76
Cancerous	120
Castration Resistant Prostate Cancer (CRPC)	8
Metastasis	17
Patient Age at Biopsy	118
Gleason Grade	67

C

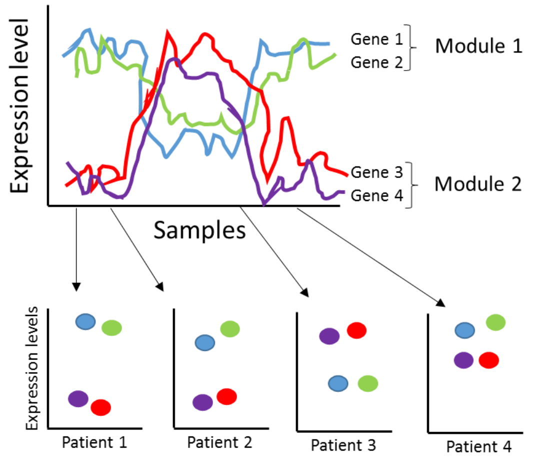

D

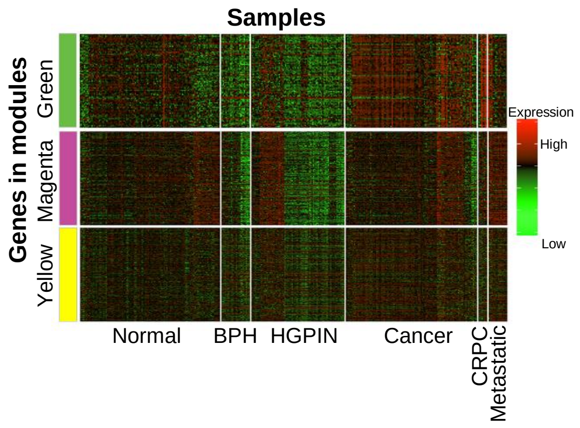

Appendix Figure S1: Meta-analysis of human gene expression profiling arrays identifies gene modules that correlate with prostate cancer progression.

(A), (B) The tables summarize the eight independent microarrays used for the WGCNA analysis, comprising 375 human prostate samples; and the different prostate phenotypes.

(C) The schematics depict the underlying concept of WGCNA in which gene modules are defined by identifying those genes whose expression changes similarly across different patients.

(D) Heat map depicts gene expression levels of *Green, Magenta* and *Yellow* modules, which significantly correlate with disease progression, and their relationships to phenotype/disease stage from WGCNA analysis.

Appendix Figure S2

A

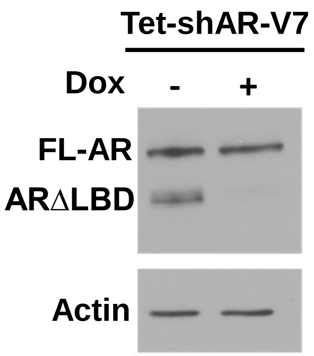

B

APOBEC3B	CCNE2	CENPM	KIF11	NUF2	TACC3
ASPM	CDC20	CEP55	KIF14	NUSAP1	TOP2A
AURKA	CDCA8	DEPDC1B	KIF18A	PBK	TPX2
BIRC5	CDK1	DLGAP5	KIF20A	RACGAP1	TYMS
BUB1	CDKN3	E2F8	KIF23	RAD51	UBE2T
BUB1B	CDT1	ECT2	KIF4A	RAD51AP1	UHRF1
CALCOCO1	CENPE	FAM49B	MAD2L1	RFC4	ZNF367
CASC5	CENPF	GART	NCAPG	RMI1	ZNF92
CCNB1	CENPI	GINS1	NCAPH	SGOL2	ZWINT
CCNB2	CENPK	HMMR	NDC1	SPC24	ZYX

C

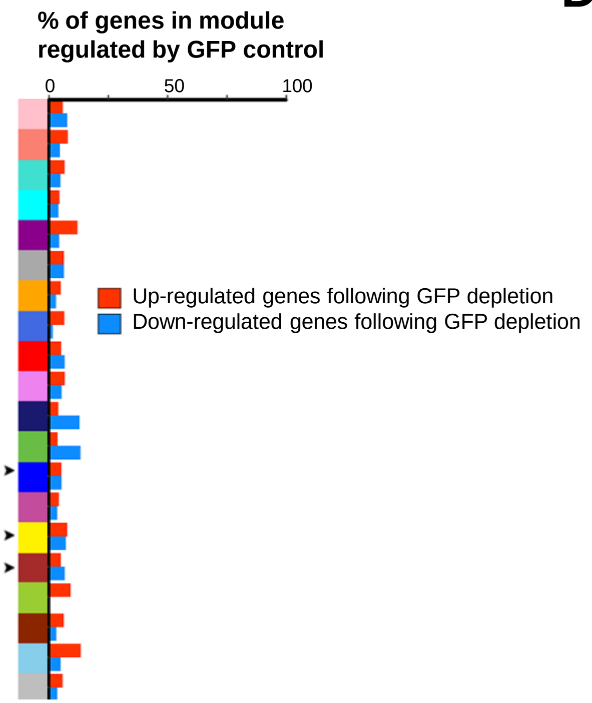

D

Rank	Enrichment P	Bonferroni P	Process
1	2.41E-41	3.83E-37	mitotic cell cycle
2	5.57E-39	8.85E-35	cell cycle
3	2.12E-37	3.36E-33	cell cycle process
4	2.94E-35	4.67E-31	mitotic cell cycle process
5	1.85E-33	2.95E-29	cell division
6	6.06E-32	9.63E-28	mitotic nuclear division
7	8.23E-28	1.31E-23	chromosome segregation
8	2.95E-22	4.69E-18	sister chromatid segregation

Appendix Figure S2: shGFP control samples for the gene expression array are not significantly enriched among WGCNA modules.

(A) 22Rv1 cells stably expressing tet-shAR-V7 were grown in 5% CSS ± doxycycline for 72 hours. Equivalent amounts of total cellular protein were immunoblotted for N-terminal AR and actin (AR(N20) Cat. Sc-816 and actin Cat. Sc-47778).

(B) List of the 60 genes in the *green* module regulated by AR-V7.

(C) Microarray analysis was performed in 22Rv1 PC cells in doxycycline-treated shGFP controls. The genes that were significantly regulated by shGFP (in either direction, p value < 0.05) were distributed among the gene modules defined by WGCNA in Figure 1a. Up-regulated genes are those in which expression increased following GFP depletion. Conversely, downregulated genes are those that decreased following GFP depletion.

(D) Results from pathway enrichment analysis of the *green* module is shown.

Appendix Figure S3

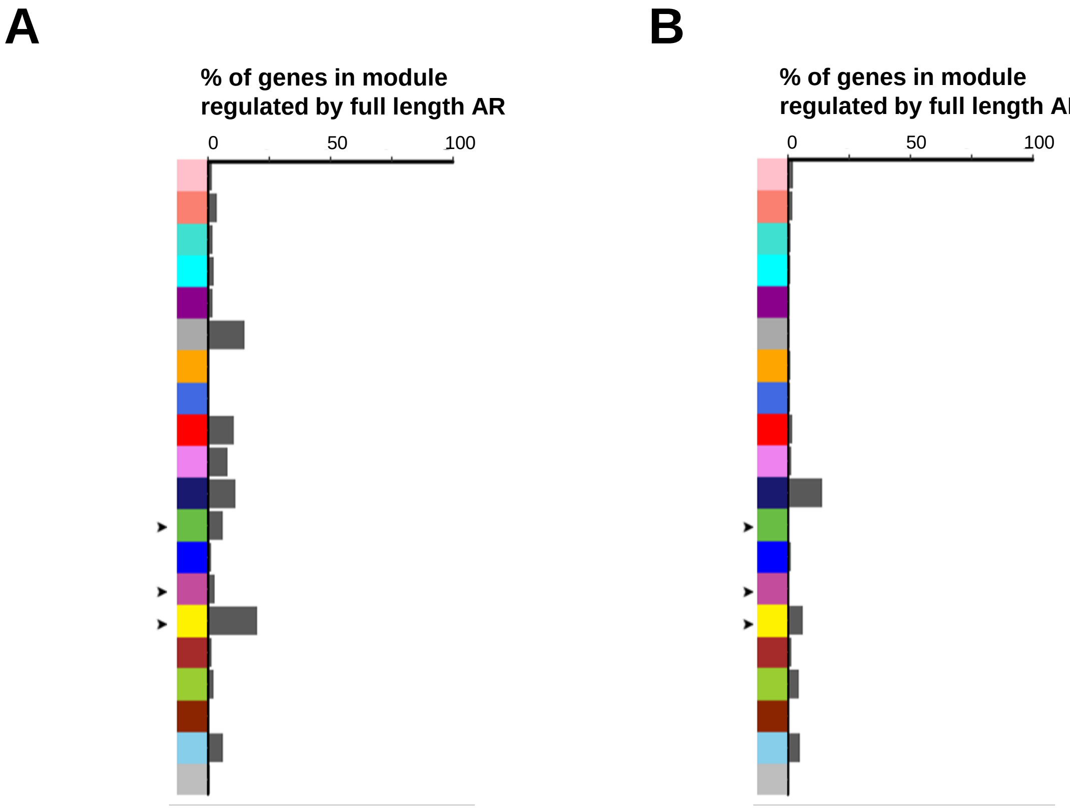

Appendix Figure S3: The green module was not enriched for full length AR regulated genes.
(A), (B) Full length AR signatures were obtained from Pomerantz et al., 2015, and Mendiratta et al., 2009. The list of full length AR regulated genes were distributed among the gene modules defined by WGCNA in Figure 1a.

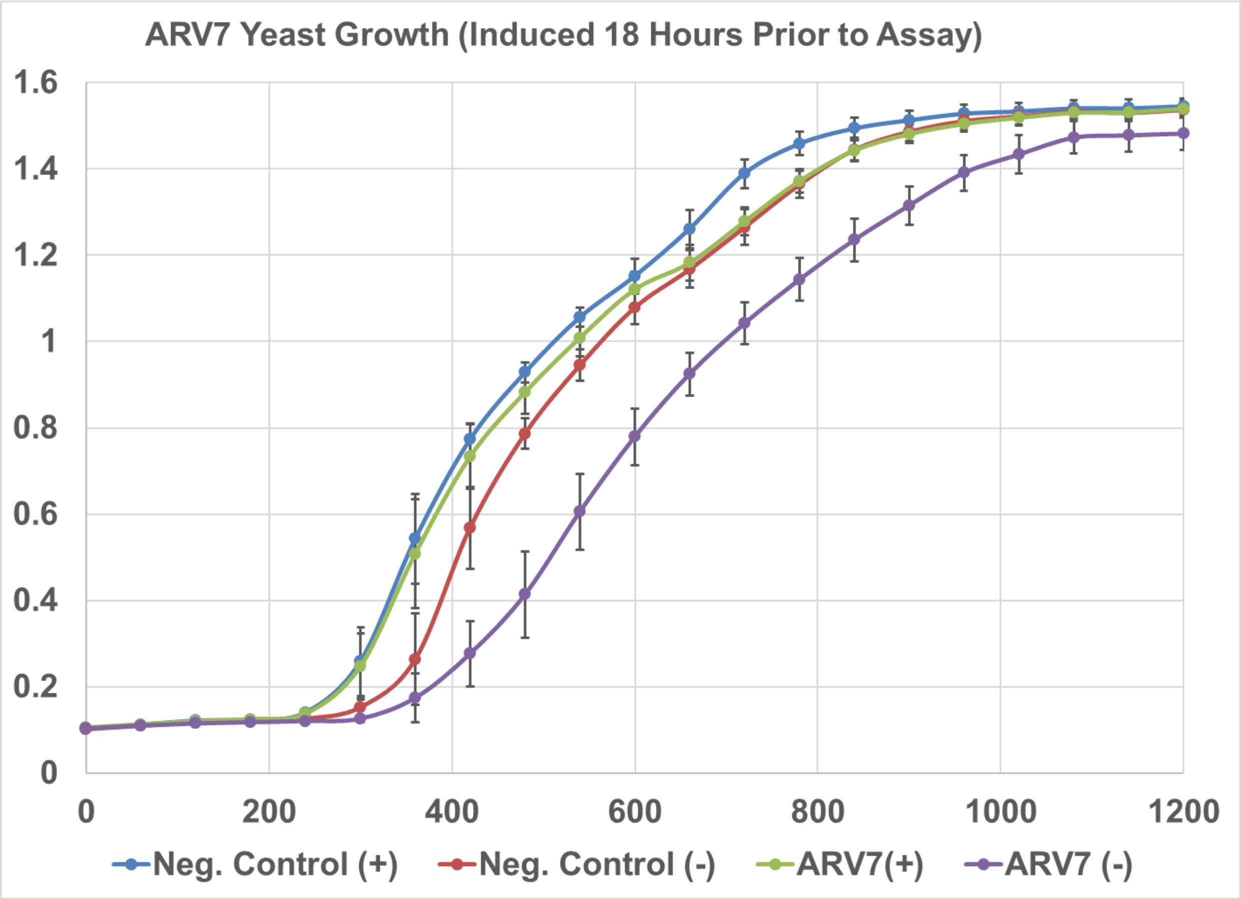

Appendix Figure S4: Expression of AR-V7 generated a slight growth defect on the yeast strain. The inducible *S. pombe* strain expressing an HA-tagged AR-V7 fusion protein was integrated under the control of the *nmt1* thiamine-repressible promoter. Growth curve analysis was completed in the presence (+) and absence of thiamine(-). Details of the procedure can be found in Wiley et al., 2014.

Appendix Figure S5

Network Stats

number of nodes:	273	expected number of edges:	209
number of edges:	358	PPI enrichment p-value:	0
average node degree:	2.62	*your network has significantly more interactions*	
clustering coefficient:	0.876	*than expected (what does that mean?)*	

Functional enrichments in your network

Biological Process (GO)

pathway ID	*pathway description*	*count in network*	*false discovery rate*
GO:0070972	protein localization to endoplasmic reticulum	25	1.24e-17
GO:0000184	nuclear-transcribed mRNA catabolic process, nonsense-mediated decay	23	7.77e-17
GO:0000956	nuclear-transcribed mRNA catabolic process	26	1.73e-16
GO:0006614	SRP-dependent cotranslational protein targeting to membrane	21	1.94e-15
GO:0019083	viral transcription	21	3.29e-15
			(more ...)

Molecular Function (GO)

pathway ID	*pathway description*	*count in network*	*false discovery rate*
GO:0003735	structural constituent of ribosome	15	1.48e-07
GO:0003824	catalytic activity	112	1.48e-07
GO:0005342	organic acid transmembrane transporter activity	14	1.48e-07
GO:0008514	organic anion transmembrane transporter activity	16	1.48e-07
GO:0008509	anion transmembrane transporter activity	18	8.06e-07
			(more ...)

Cellular Component (GO)

pathway ID	*pathway description*	*count in network*	*false discovery rate*
GO:0022626	cytosolic ribosome	22	5.51e-19
GO:0044445	cytosolic part	24	3.47e-15
GO:0044391	ribosomal subunit	21	1.86e-14
GO:0044422	organelle part	160	2.3e-13
GO:0022625	cytosolic large ribosomal subunit	14	2.95e-13
			(more ...)

KEGG Pathways

pathway ID	*pathway description*	*count in network*	*false discovery rate*
03010	Ribosome	21	9.76e-15
04114	Oocyte meiosis	15	1.41e-09
04110	Cell cycle	15	7.54e-09
05203	Viral carcinogenesis	13	7.23e-05
05034	Alcoholism	11	0.000135
			(more ...)

PFAM Protein Domains

pathway ID	*pathway description*	*count in network*	*false discovery rate*
PF00125	Core histone H2A/H2B/H3/H4	12	3.11e-08
PF00244	14-3-3 protein	6	5.53e-08
PF00149	Calcineurin-like phosphoesterase	4	0.0372
PF07690	Major Facilitator Superfamily	4	0.0372
PF00481	Protein phosphatase 2C	3	0.0442
			(more ...)

INTERPRO Protein Domains and Features

pathway ID	*pathway description*	*count in network*	*false discovery rate*
IPR002119	Histone H2A	12	1.9e-13
IPR007125	Histone H2A/H2B/H3	12	3.32e-08
IPR000308	14-3-3 protein	6	4.73e-08
IPR023409	14-3-3 protein, conserved site	6	4.73e-08
IPR023410	14-3-3 domain	6	4.73e-08
			(more ...)

Appendix Figure S5: Network analysis of AR-V7 interactome was obtained using STRING of hits identified by SGA screening.

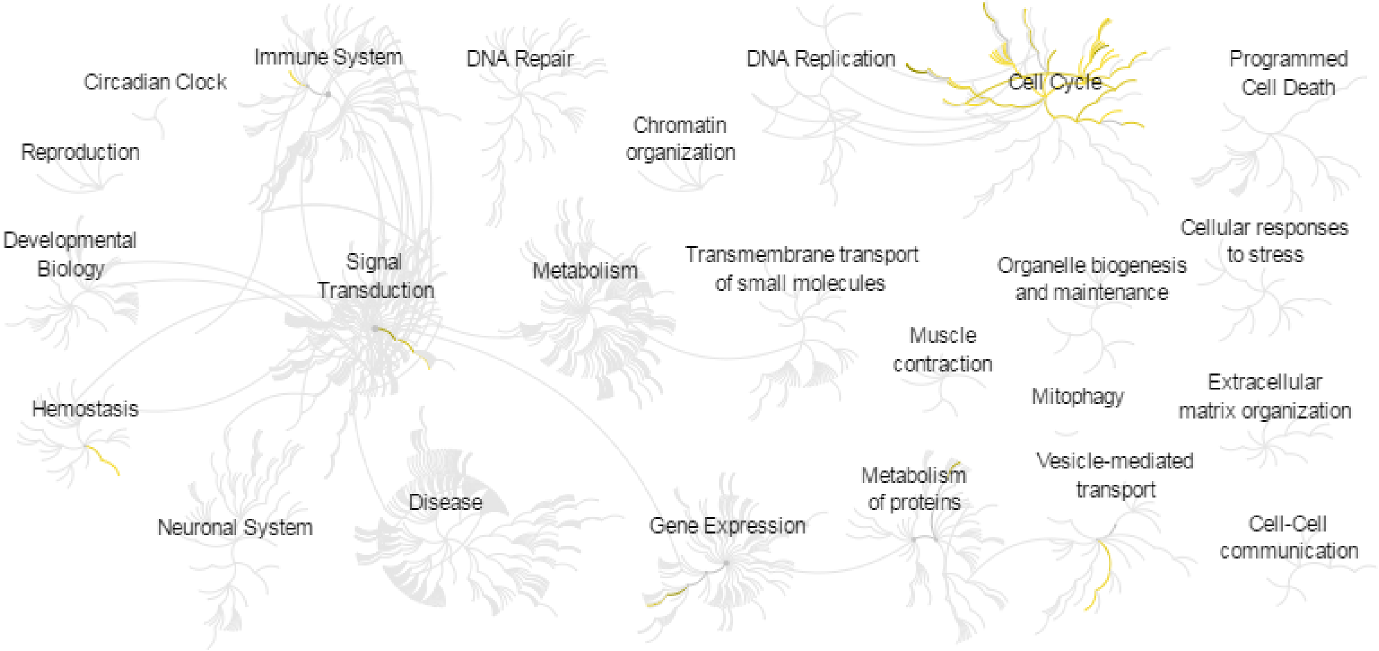

Appendix Figure S6: Pathway analysis of the seven gene set reveals a strong association to cell cycle. The analysis was performed using *https://reactome.org.*

A

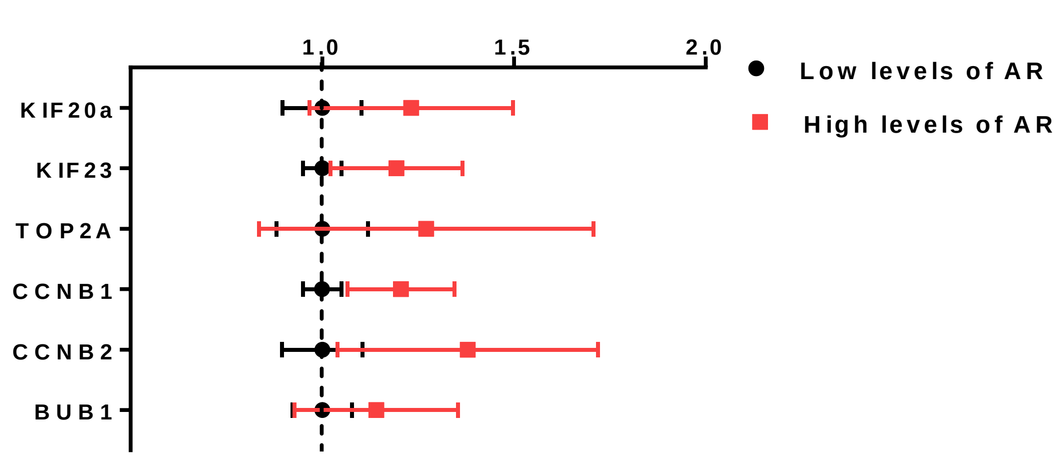

B

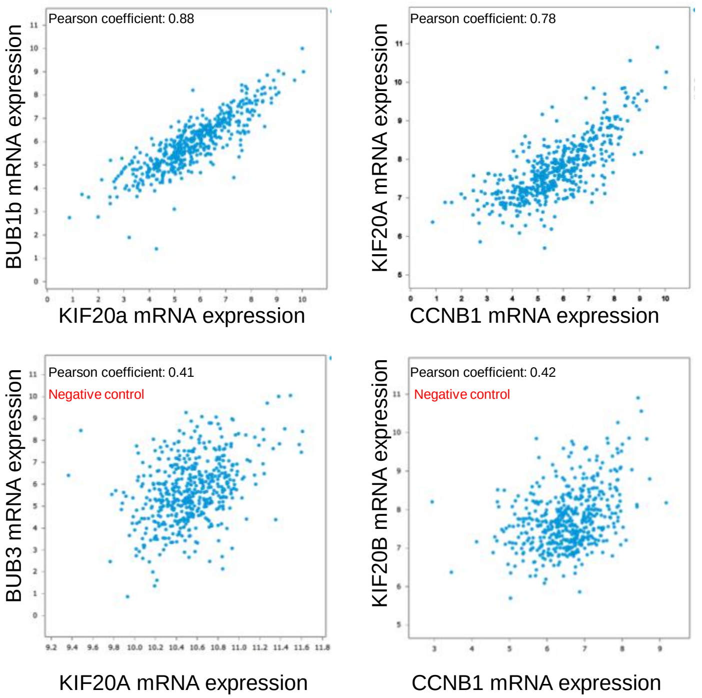

Gene 1	Gene 2	Pearson's Correlation	Gene 1	Gene 2	Pearson's Correlation
KIF20A	KIF23	0.90	TOP2A	CCNB1	0.75
KIF20A	TOP2A	0.87	TOP2A	CCNB2	0.66
KIF20A	CCNB1	0.78	TOP2A	BUB1	0.92
KIF20A	CCNB2	0.70	TOP2A	BUB1B	0.85
KIF20A	BUB1	0.86	CCNB1	CCNB2	0.68
KIF20A	BUB1B	0.88	CCNB1	BUB1	0.80
KIF23	TOP2A	0.92	CCNB1	BUB1B	0.74
KIF23	CCNB1	0.80	CCNB2	BUB1	0.70
KIF23	CCNB2	0.71	CCNB2	BUB1B	0.70
KIF23	BUB1	0.92	BUB1	BUB1B	0.88
KIF23	BUB1B	0.88			

Appendix Figure S7: The seven genes are co-expressed in human PC samples, but do not correlate with full length AR expression levels.

(A) Hornberg *et al*., 2011 gene expression profiling array data was analyzed to determine the expression levels of the seven genes in human CRPC bone metastases, grouped by their relative levels of AR (High-levels of AR (top quartile) or lower levels of AR (quartiles 1-3). Data are plotted as the mean ± s.e.m.. Non-parametric Mann-Whitney test was performed (two-tailed). Note that BUB1B expression was not measured in these microarrays.

(B) The left panels (top graphs) show sample pairwise comparisons (KIF20a & BUB1B; CCNB1 & KIF20a mRNAs) in human PC samples obtained from the TCGA Provisional Adenocarcinoma dataset, where log2 transformation was applied (n= 499). Analysis of BUB3 and KIF20b, which are not members of the seven gene set, were compared with KIF20A and CCNB1, respectively, as negative controls. The table summarizes the Pearson correlation values between the expression levels of each pair of the seven genes.

Appendix Figure S8

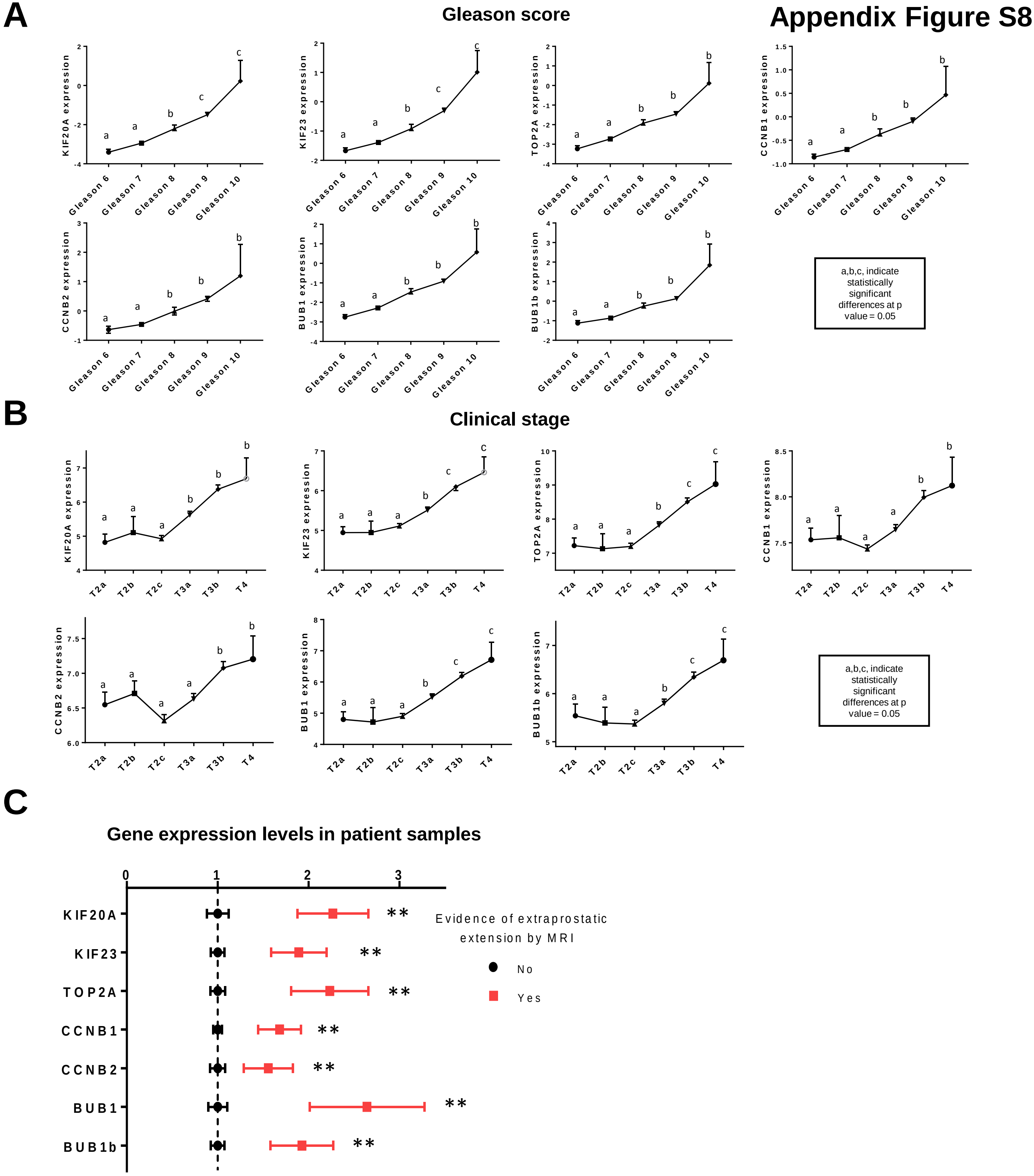

Appendix Figure S8: Elevated expression of the seven gene set is associated with higher Gleason scores and other adverse indicators in human PC samples.

(A), **(B)** PC patient RNA-SEQ data from the Prostate Adenocarcinoma TCGA provisional dataset (N = 465) were used to plot the log2 based transformed mean + s.e.m. of the gene expression levels for each PC sample according to each patient's Gleason score and clinical stage. Kruskal-Wallis (P value<0.0001 for all panels, two-tailed) and Dunn's multiple comparisons test were performed. Different letters (a,b,c) denote statistically significant differences at a p value < 0.05.

(C) PC patient RNA-seq data from the Prostate Adenocarcinoma TCGA provisional were analyzed to compare gene expression levels with the presence or absence of evidence for extraprostatic extension by MRI analysis (an adverse prognostic indicator). Data shows the mean ± s.e.m., Unpaired t-test with Welch's correction were performed (two-tailed). ** Significant at a p value < 0.05.

Appendix Figure S9

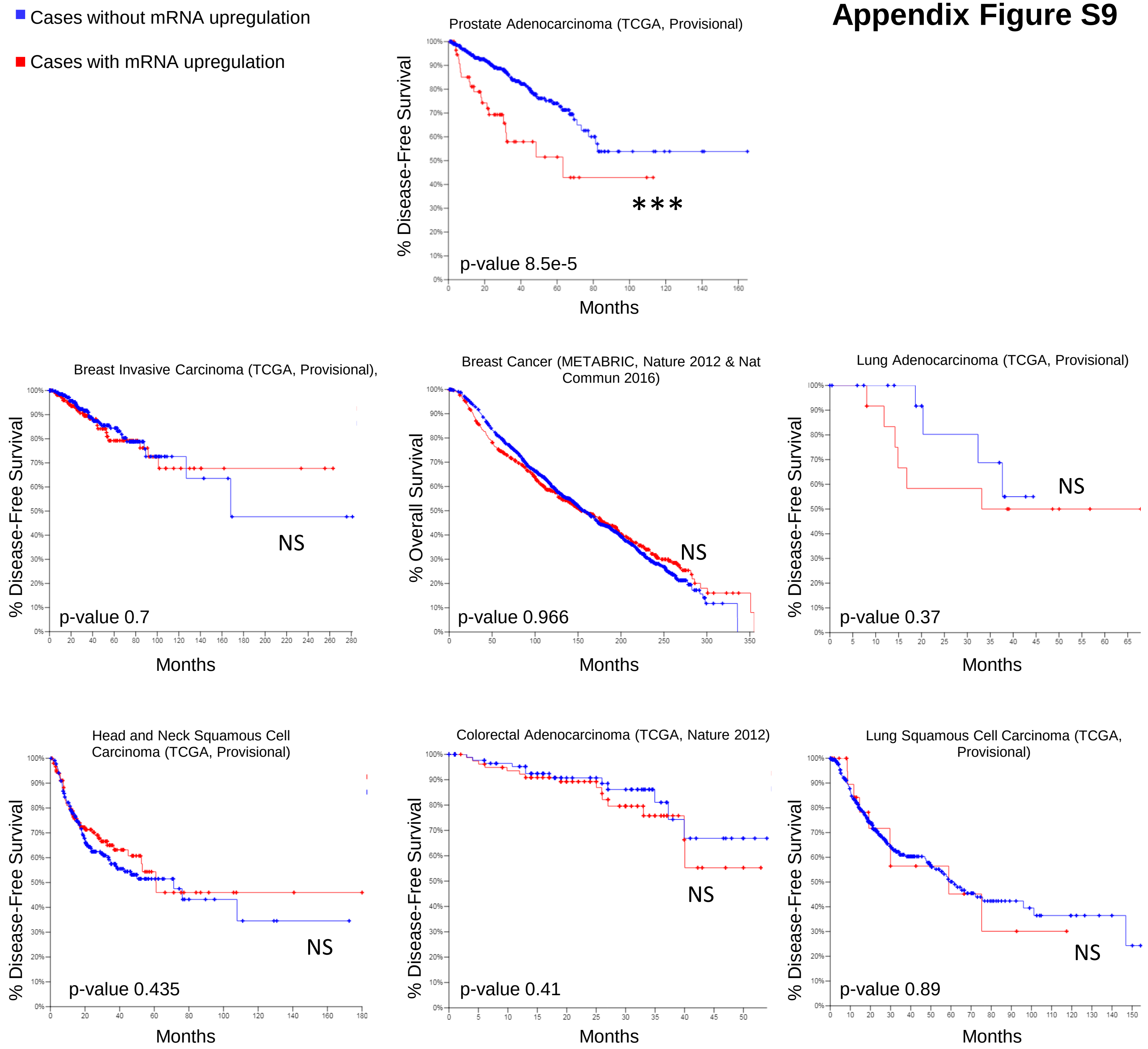

Appendix Figure S9: The seven gene set does not have prognostic value in several other types of cancer. The Kaplan-Meier curves for Disease-Free Survival were built with cbioportal.org, using the following datasets: Prostate Cancer Adenocarcinoma (TCGA, Provisional), Breast Invasive Carcinoma (TCGA, Provisional), Breast Cancer (METABRIC, Nature 2012 & Nat Commun 2016), Lung Adenocarcinoma (TCGA, Provisional), Head and Neck Squamous Cell Carcinoma (TCGA, Provisional), Colorectal Adenocarcinoma (TCGA, Nature 2012), and Lung Squamous Cell Carcinoma (TCGA, Provisional). The blue curve denotes cases with normal expression of the gene set, and red represents cases where the mRNA levels of one or more of the seven genes were upregulated (z-score threshold ± 1.5).

Appendix Figure S10

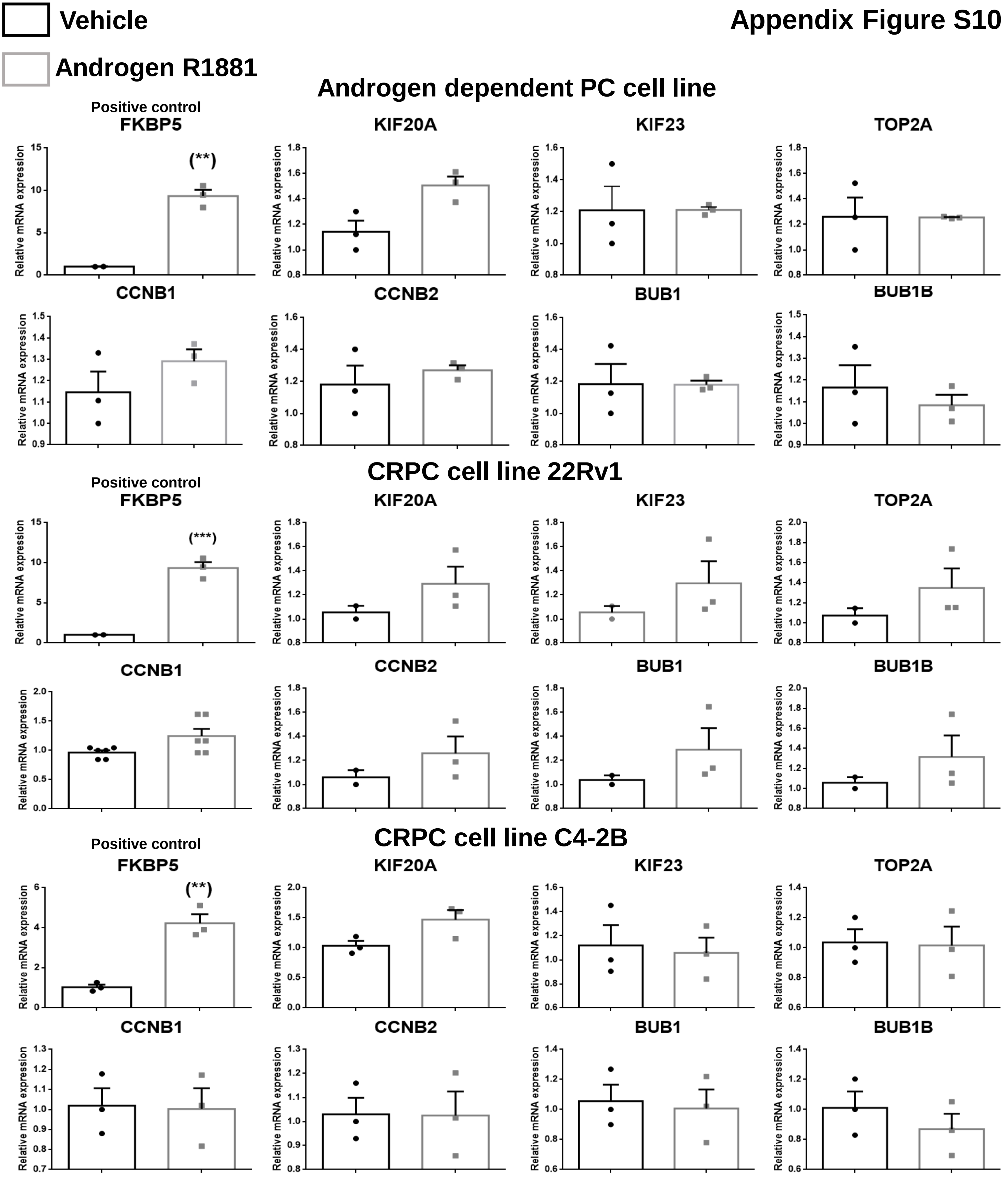

Appendix Figure S10: None of the seven genes are regulated by ligand-activated full length AR.
LNCaP, 22Rv1, and C4-2B cells were seeded and 24 hours later were rinsed twice with PBS and incubated in serum free media for one hour. The cells were then incubated in media containing vehicle or 0.1 nM of R1881 for 16 hours prior to harvesting them for RNA extraction and qPCR analysis. FKBP5 was used as a positive control. Data represent one to two independent experiments, performed in biological triplicates, showing the mean ± s.e.m., and normalized to vehicle control. Significant at a p value, ** p value < 0.01, *** p value < 0.001.

Appendix Figure S11

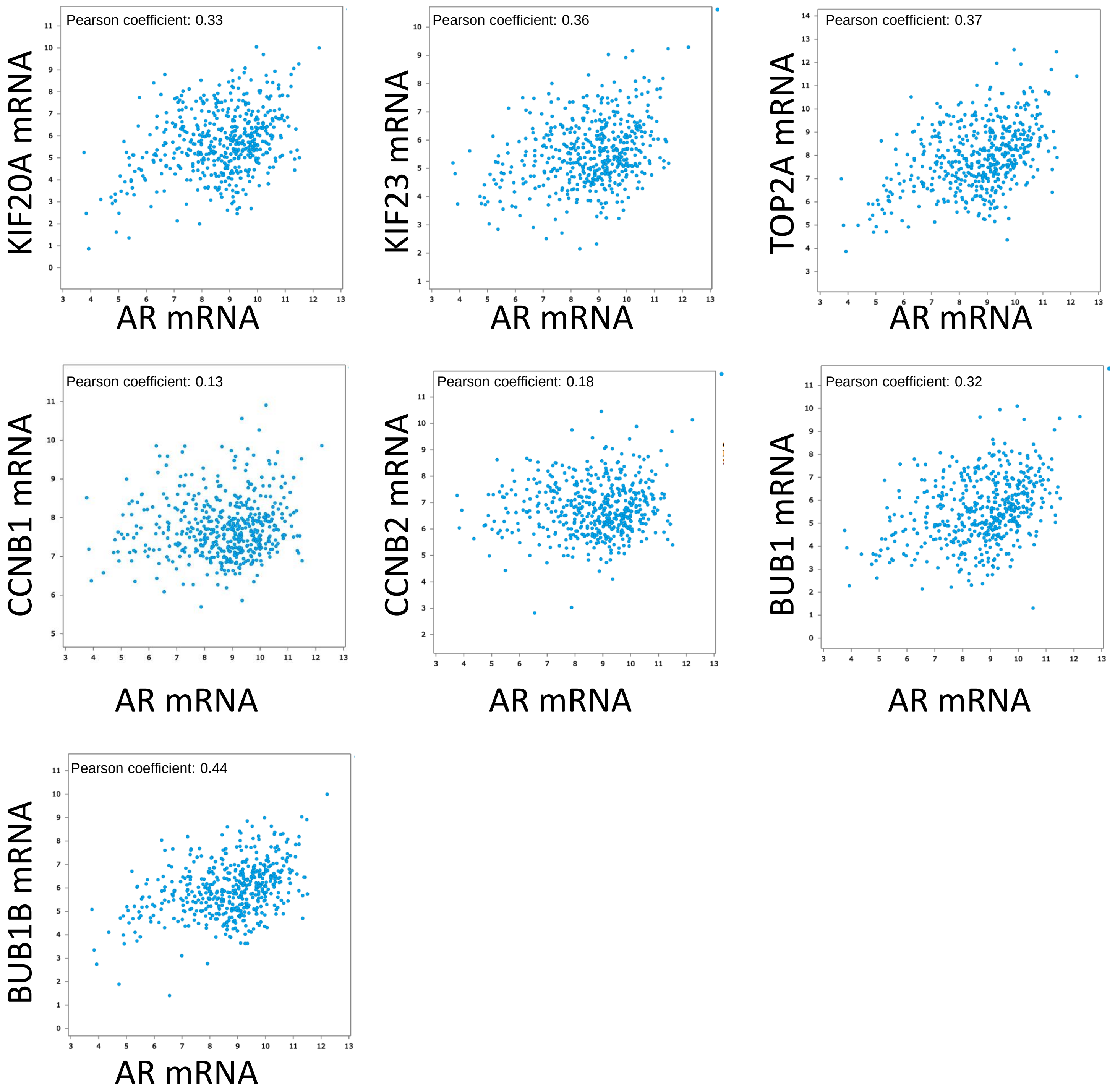

Appendix Figure S11: The seven gene set is not associated to full length AR levels in human samples. The graphs show pairwise comparisons of the mRNA levels of each of the seven genes with the mRNA levels of AR in human PC samples obtained from the TCGA Provisional Adenocarcinoma dataset, where log2 transformation was applied.

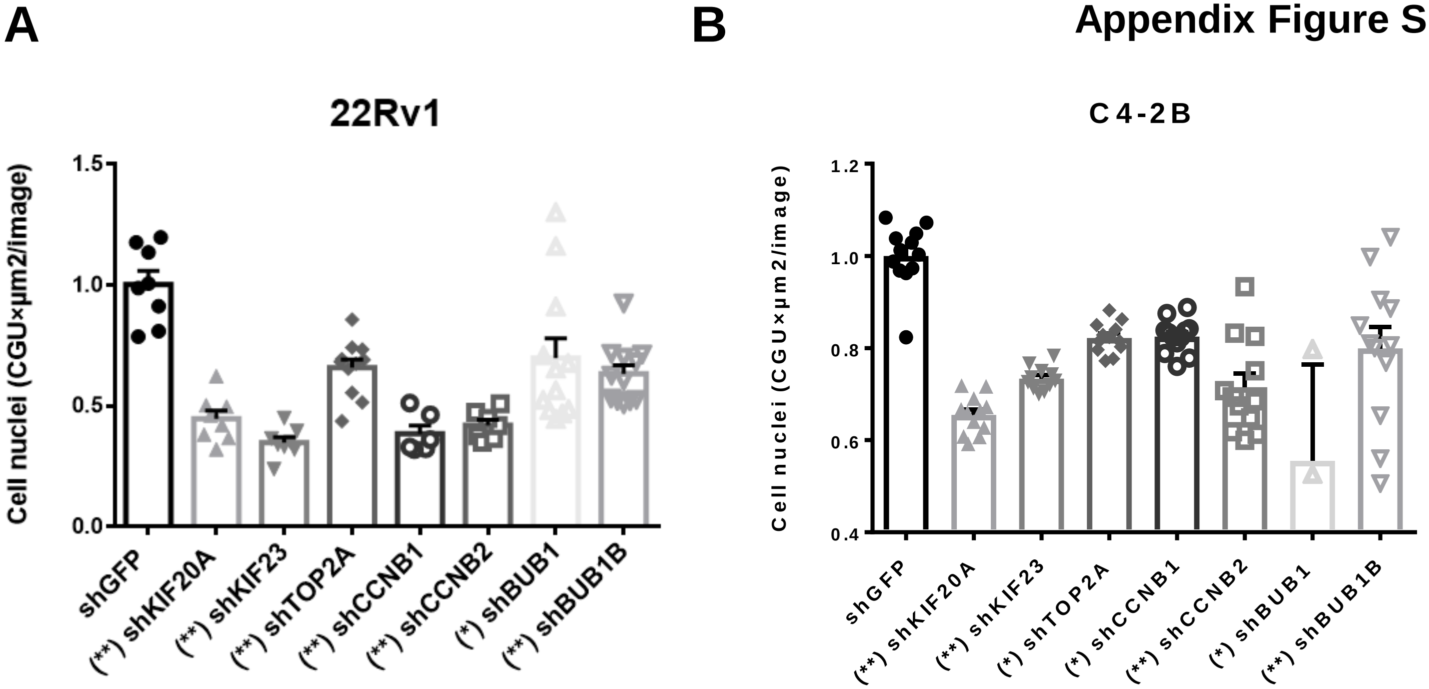

Appendix Figure S12: Stable depletion of the expression of each of the seven genes reduces CRPC cell proliferation in two different cell lines.

(A), (B) Cell proliferation was examined in the CRPC cell lines 22Rv1 and C4-2B following individual depletion of mRNAs for the seven genes or shGFP controls. shRNA constructs against the 3'UTR of each gene were used. Cell number was measured using a non-perturbing nuclear-restricted dye and quantified after 72 hours using Incucyte Zoom System. Data shown are mean ± s.e.m. of 8 to 12 replicates normalized to their shGFP control. Kruskal-Wallis test (p value < 0.0001, two-tailed) and Dunn's multiple comparisons test were performed.

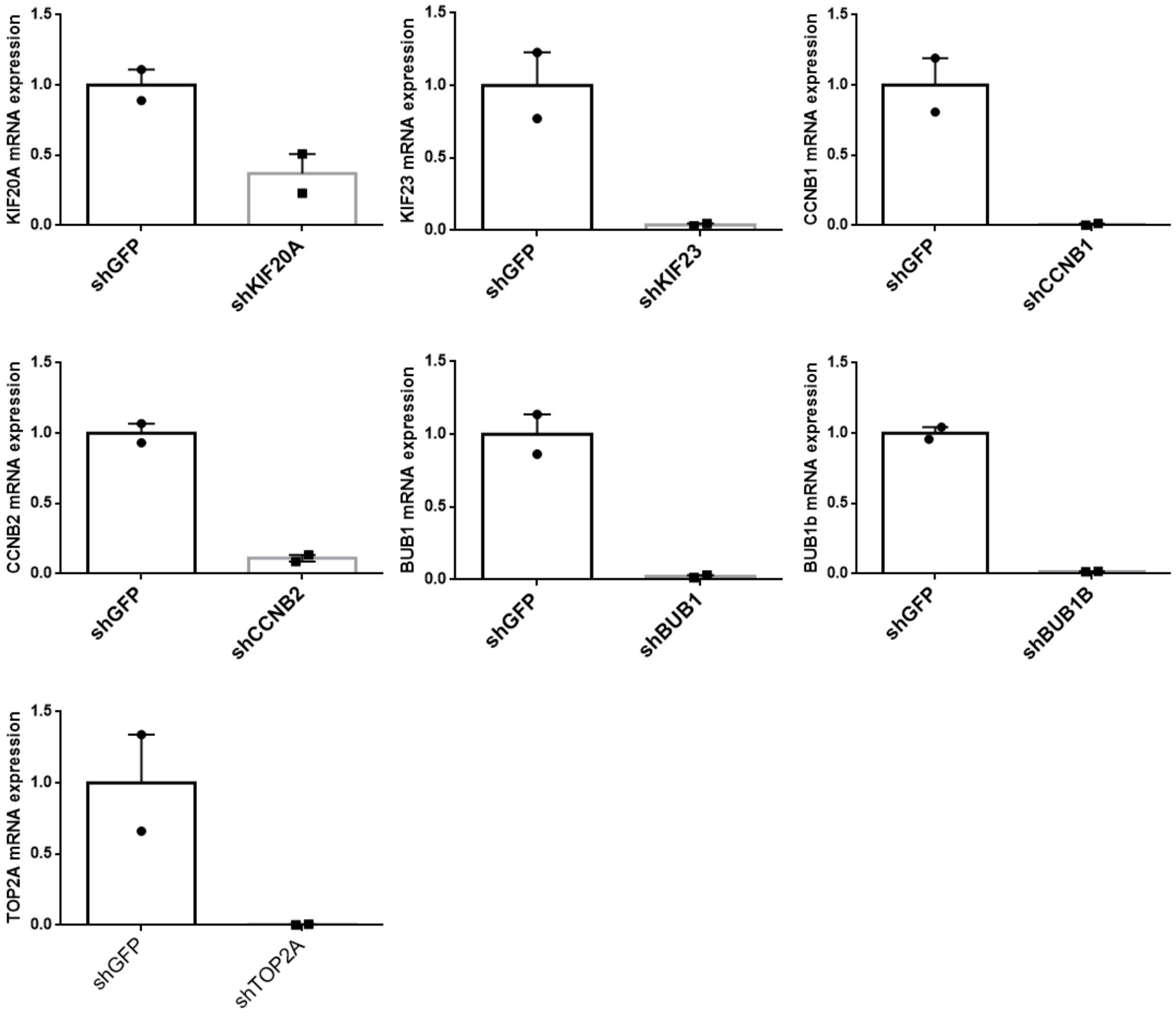

Appendix Figure S13: Analysis of efficacy of shRNA-mediated depletion of the expression of each of the seven genes.

22Rv1 cells were stably transduced with shGFP (as a control) or shRNAs targeted to each of the seven genes. RT-qPCR analysis was performed in duplicate, and the results were normalized to GAPDH mRNA levels, and then to the respective shGFP controls. The median with 95% CI is shown.

Appendix Figure S14

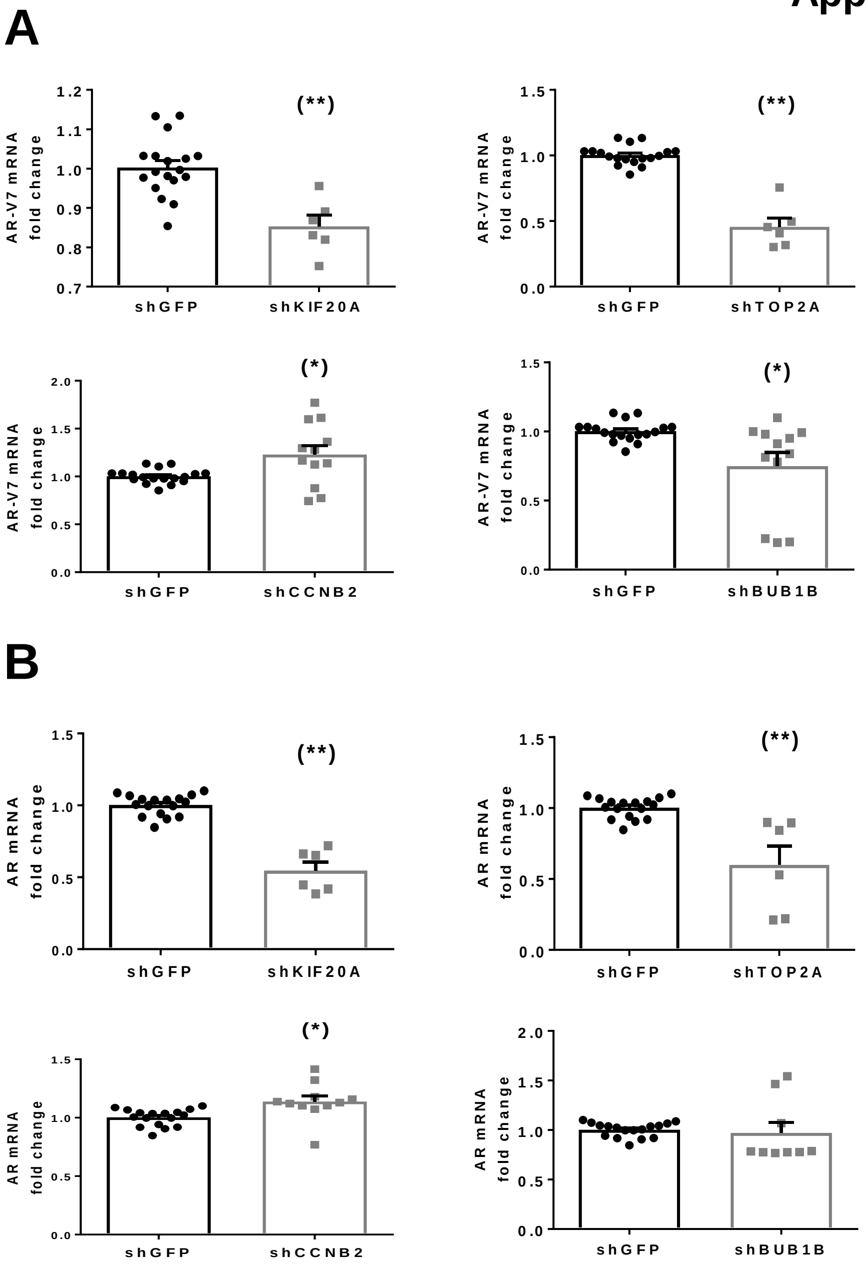

Appendix Figure S14: Members of the gene set modify AR-V7 and full length AR expression levels.
(A), (B) 22Rv1 cells were stably transduced with shGFP (as a control) or shRNAs targeted to individual members of the seven genes. Cells were kept in 2% CSS. mRNA was harvested and RT-qPCR analysis was performed to measure AR-V7 or full length AR mRNA levels, in triplicates two to four independent times. The results were normalized to GAPDH mRNA levels, and then to the respective shGFP controls. Data shows the mean ± s.e.m. Unpaired t-test with Welch's correction were performed (two-tailed). Significant at a p value, * p value < 0.05, ** p value < 0.01.

Appendix Figure S15

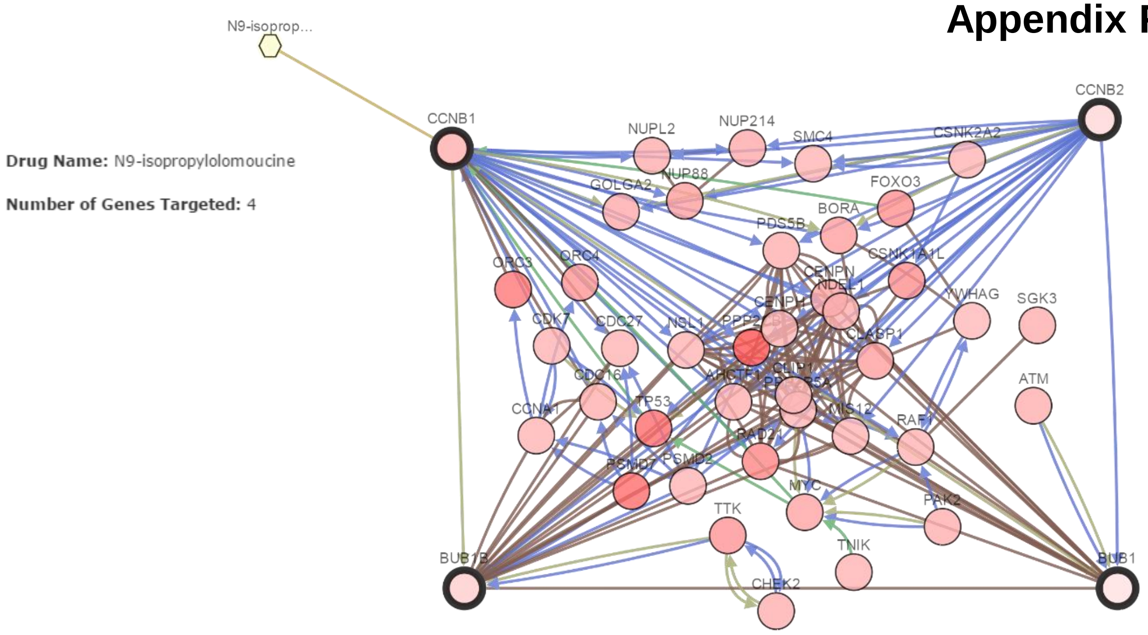

Appendix Figure S15: The compound N-9 affects directly and indirectly four of the seven genes due to pathway interactions.

Network interactions were mapped using cbioportal.org as described in Figure 1E legend. The type of gene to gene interactions as defined by cbioportal.org are: controls state change of (blue), controls expression of (green), in complex with (brown), drug target (yellow).
