# Supplementary material for: Identification of an oncogenic network with prognostic and therapeutic value in prostate cancer
Source: Mol Syst Biol. 2018 Aug 14;14(8):e8202. doi: 10.15252/msb.20188202 (PMC6684952; doi:10.15252/msb.20188202)
Supplement: Supplementary file 4 — Source Data for Figure 1C [file MSB-14-e8202-s002.pptx]

## Slide 1
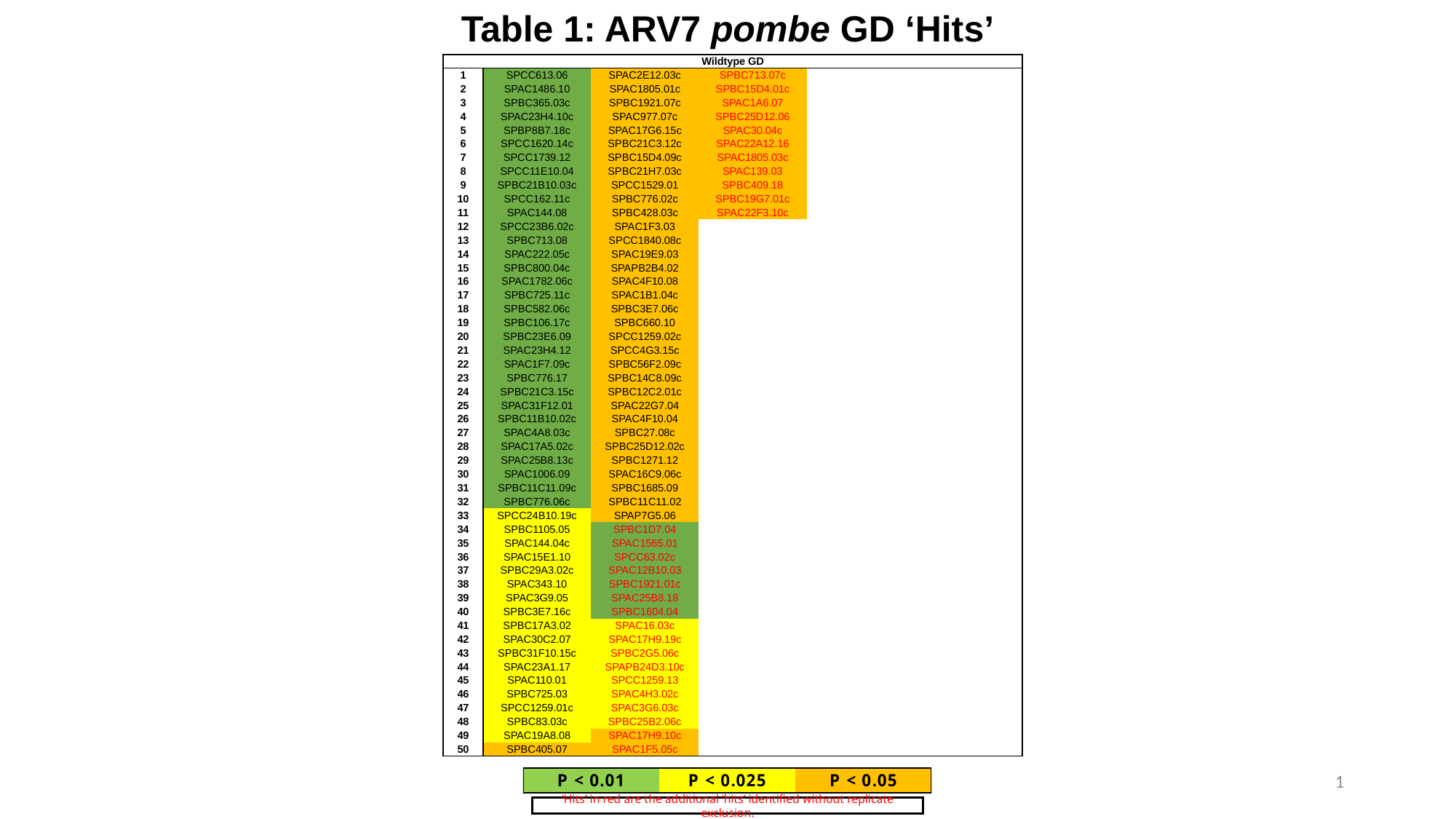

Table 1: ARV7 pombe GD ‘Hits’
| Wildtype GD | | | | | |
| --- | --- | --- | --- | --- | --- |
| 1 | SPCC613.06 | SPAC2E12.03c | SPBC713.07c | | |
| 2 | SPAC1486.10 | SPAC1805.01c | SPBC15D4.01c | | |
| 3 | SPBC365.03c | SPBC1921.07c | SPAC1A6.07 | | |
| 4 | SPAC23H4.10c | SPAC977.07c | SPBC25D12.06 | | |
| 5 | SPBP8B7.18c | SPAC17G6.15c | SPAC30.04c | | |
| 6 | SPCC1620.14c | SPBC21C3.12c | SPAC22A12.16 | | |
| 7 | SPCC1739.12 | SPBC15D4.09c | SPAC1805.03c | | |
| 8 | SPCC11E10.04 | SPBC21H7.03c | SPAC139.03 | | |
| 9 | SPBC21B10.03c | SPCC1529.01 | SPBC409.18 | | |
| 10 | SPCC162.11c | SPBC776.02c | SPBC19G7.01c | | |
| 11 | SPAC144.08 | SPBC428.03c | SPAC22F3.10c | | |
| 12 | SPCC23B6.02c | SPAC1F3.03 | | | |
| 13 | SPBC713.08 | SPCC1840.08c | | | |
| 14 | SPAC222.05c | SPAC19E9.03 | | | |
| 15 | SPBC800.04c | SPAPB2B4.02 | | | |
| 16 | SPAC1782.06c | SPAC4F10.08 | | | |
| 17 | SPBC725.11c | SPAC1B1.04c | | | |
| 18 | SPBC582.06c | SPBC3E7.06c | | | |
| 19 | SPBC106.17c | SPBC660.10 | | | |
| 20 | SPBC23E6.09 | SPCC1259.02c | | | |
| 21 | SPAC23H4.12 | SPCC4G3.15c | | | |
| 22 | SPAC1F7.09c | SPBC56F2.09c | | | |
| 23 | SPBC776.17 | SPBC14C8.09c | | | |
| 24 | SPBC21C3.15c | SPBC12C2.01c | | | |
| 25 | SPAC31F12.01 | SPAC22G7.04 | | | |
| 26 | SPBC11B10.02c | SPAC4F10.04 | | | |
| 27 | SPAC4A8.03c | SPBC27.08c | | | |
| 28 | SPAC17A5.02c | SPBC25D12.02c | | | |
| 29 | SPAC25B8.13c | SPBC1271.12 | | | |
| 30 | SPAC1006.09 | SPAC16C9.06c | | | |
| 31 | SPBC11C11.09c | SPBC1685.09 | | | |
| 32 | SPBC776.06c | SPBC11C11.02 | | | |
| 33 | SPCC24B10.19c | SPAP7G5.06 | | | |
| 34 | SPBC1105.05 | SPBC1D7.04 | | | |
| 35 | SPAC144.04c | SPAC1565.01 | | | |
| 36 | SPAC15E1.10 | SPCC63.02c | | | |
| 37 | SPBC29A3.02c | SPAC12B10.03 | | | |
| 38 | SPAC343.10 | SPBC1921.01c | | | |
| 39 | SPAC3G9.05 | SPAC25B8.18 | | | |
| 40 | SPBC3E7.16c | SPBC1604.04 | | | |
| 41 | SPBC17A3.02 | SPAC16.03c | | | |
| 42 | SPAC30C2.07 | SPAC17H9.19c | | | |
| 43 | SPBC31F10.15c | SPBC2G5.06c | | | |
| 44 | SPAC23A1.17 | SPAPB24D3.10c | | | |
| 45 | SPAC110.01 | SPCC1259.13 | | | |
| 46 | SPBC725.03 | SPAC4H3.02c | | | |
| 47 | SPCC1259.01c | SPAC3G6.03c | | | |
| 48 | SPBC83.03c | SPBC25B2.06c | | | |
| 49 | SPAC19A8.08 | SPAC17H9.10c | | | |
| 50 | SPBC405.07 | SPAC1F5.05c | | | |
1
‘Hits’ in red are the additional ‘hits’ identified without replicate exclusion.

## Slide 2
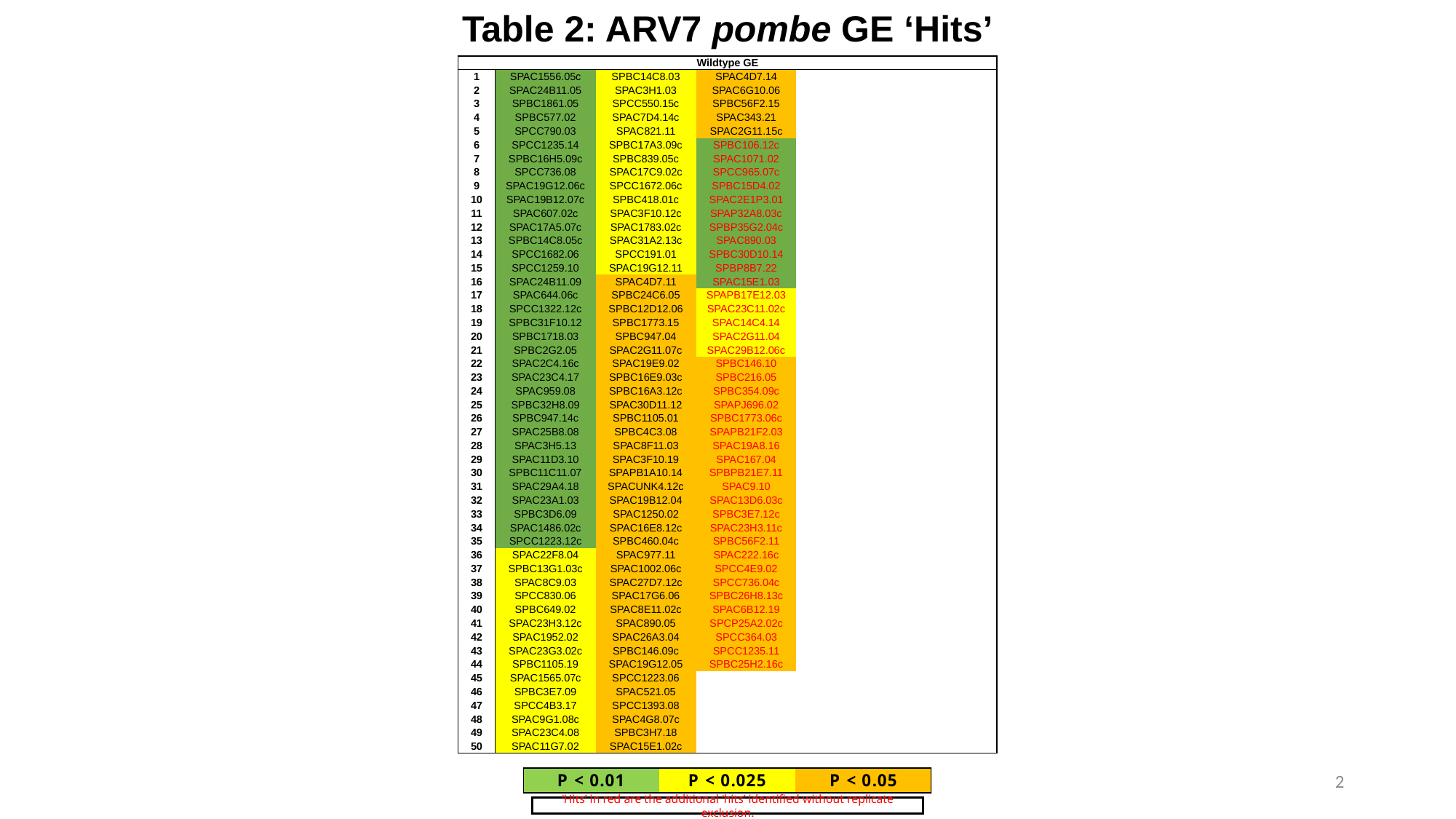

Table 2: ARV7 pombe GE ‘Hits’
| Wildtype GE | | | | | |
| --- | --- | --- | --- | --- | --- |
| 1 | SPAC1556.05c | SPBC14C8.03 | SPAC4D7.14 | | |
| 2 | SPAC24B11.05 | SPAC3H1.03 | SPAC6G10.06 | | |
| 3 | SPBC1861.05 | SPCC550.15c | SPBC56F2.15 | | |
| 4 | SPBC577.02 | SPAC7D4.14c | SPAC343.21 | | |
| 5 | SPCC790.03 | SPAC821.11 | SPAC2G11.15c | | |
| 6 | SPCC1235.14 | SPBC17A3.09c | SPBC106.12c | | |
| 7 | SPBC16H5.09c | SPBC839.05c | SPAC1071.02 | | |
| 8 | SPCC736.08 | SPAC17C9.02c | SPCC965.07c | | |
| 9 | SPAC19G12.06c | SPCC1672.06c | SPBC15D4.02 | | |
| 10 | SPAC19B12.07c | SPBC418.01c | SPAC2E1P3.01 | | |
| 11 | SPAC607.02c | SPAC3F10.12c | SPAP32A8.03c | | |
| 12 | SPAC17A5.07c | SPAC1783.02c | SPBP35G2.04c | | |
| 13 | SPBC14C8.05c | SPAC31A2.13c | SPAC890.03 | | |
| 14 | SPCC1682.06 | SPCC191.01 | SPBC30D10.14 | | |
| 15 | SPCC1259.10 | SPAC19G12.11 | SPBP8B7.22 | | |
| 16 | SPAC24B11.09 | SPAC4D7.11 | SPAC15E1.03 | | |
| 17 | SPAC644.06c | SPBC24C6.05 | SPAPB17E12.03 | | |
| 18 | SPCC1322.12c | SPBC12D12.06 | SPAC23C11.02c | | |
| 19 | SPBC31F10.12 | SPBC1773.15 | SPAC14C4.14 | | |
| 20 | SPBC1718.03 | SPBC947.04 | SPAC2G11.04 | | |
| 21 | SPBC2G2.05 | SPAC2G11.07c | SPAC29B12.06c | | |
| 22 | SPAC2C4.16c | SPAC19E9.02 | SPBC146.10 | | |
| 23 | SPAC23C4.17 | SPBC16E9.03c | SPBC216.05 | | |
| 24 | SPAC959.08 | SPBC16A3.12c | SPBC354.09c | | |
| 25 | SPBC32H8.09 | SPAC30D11.12 | SPAPJ696.02 | | |
| 26 | SPBC947.14c | SPBC1105.01 | SPBC1773.06c | | |
| 27 | SPAC25B8.08 | SPBC4C3.08 | SPAPB21F2.03 | | |
| 28 | SPAC3H5.13 | SPAC8F11.03 | SPAC19A8.16 | | |
| 29 | SPAC11D3.10 | SPAC3F10.19 | SPAC167.04 | | |
| 30 | SPBC11C11.07 | SPAPB1A10.14 | SPBPB21E7.11 | | |
| 31 | SPAC29A4.18 | SPACUNK4.12c | SPAC9.10 | | |
| 32 | SPAC23A1.03 | SPAC19B12.04 | SPAC13D6.03c | | |
| 33 | SPBC3D6.09 | SPAC1250.02 | SPBC3E7.12c | | |
| 34 | SPAC1486.02c | SPAC16E8.12c | SPAC23H3.11c | | |
| 35 | SPCC1223.12c | SPBC460.04c | SPBC56F2.11 | | |
| 36 | SPAC22F8.04 | SPAC977.11 | SPAC222.16c | | |
| 37 | SPBC13G1.03c | SPAC1002.06c | SPCC4E9.02 | | |
| 38 | SPAC8C9.03 | SPAC27D7.12c | SPCC736.04c | | |
| 39 | SPCC830.06 | SPAC17G6.06 | SPBC26H8.13c | | |
| 40 | SPBC649.02 | SPAC8E11.02c | SPAC6B12.19 | | |
| 41 | SPAC23H3.12c | SPAC890.05 | SPCP25A2.02c | | |
| 42 | SPAC1952.02 | SPAC26A3.04 | SPCC364.03 | | |
| 43 | SPAC23G3.02c | SPBC146.09c | SPCC1235.11 | | |
| 44 | SPBC1105.19 | SPAC19G12.05 | SPBC25H2.16c | | |
| 45 | SPAC1565.07c | SPCC1223.06 | | | |
| 46 | SPBC3E7.09 | SPAC521.05 | | | |
| 47 | SPCC4B3.17 | SPCC1393.08 | | | |
| 48 | SPAC9G1.08c | SPAC4G8.07c | | | |
| 49 | SPAC23C4.08 | SPBC3H7.18 | | | |
| 50 | SPAC11G7.02 | SPAC15E1.02c | | | |
2
‘Hits’ in red are the additional ‘hits’ identified without replicate exclusion.

## Slide 3
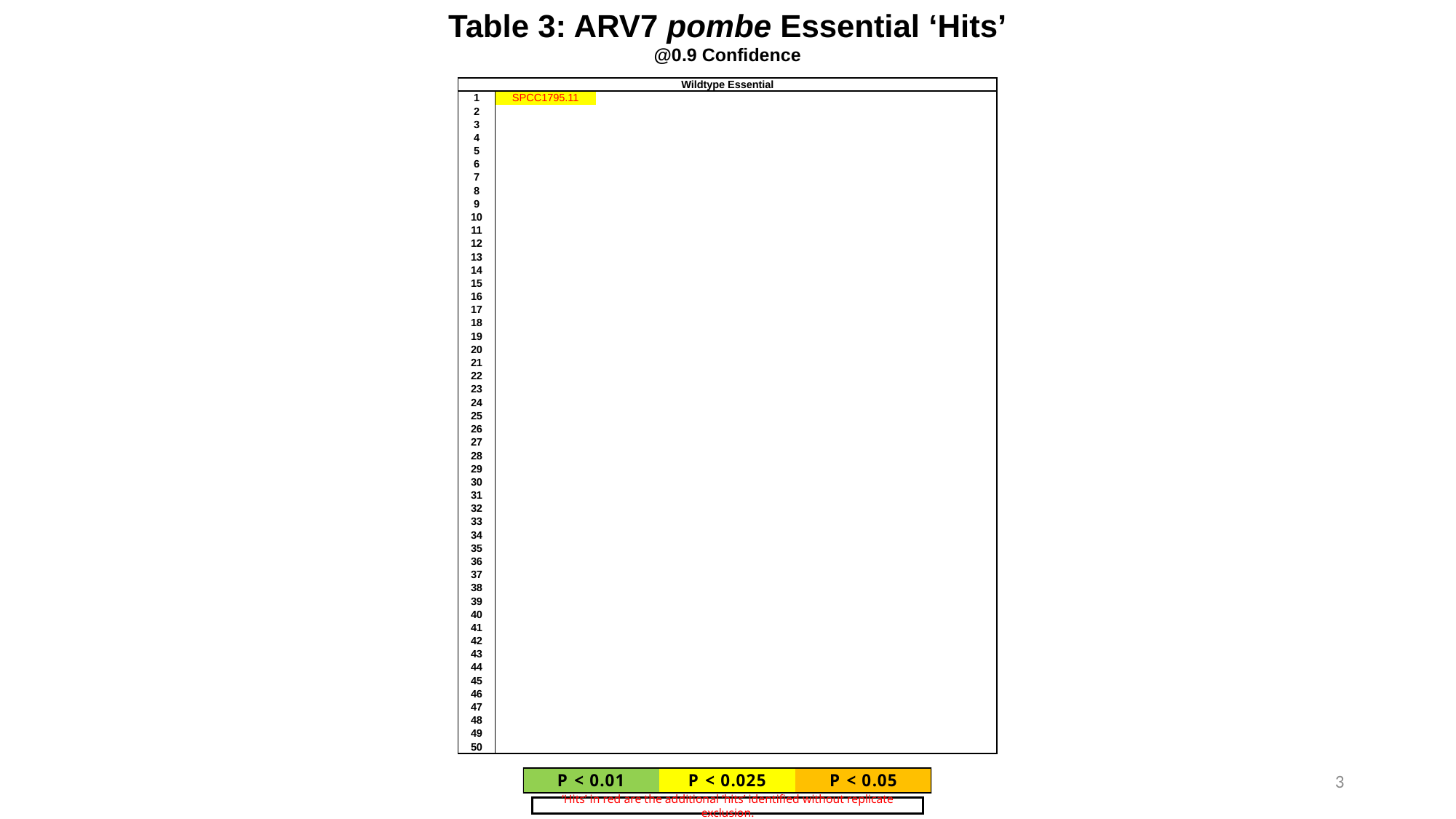

Table 3: ARV7 pombe Essential ‘Hits’
@0.9 Confidence
| Wildtype Essential | | | | | |
| --- | --- | --- | --- | --- | --- |
| 1 | SPCC1795.11 | | | | |
| 2 | | | | | |
| 3 | | | | | |
| 4 | | | | | |
| 5 | | | | | |
| 6 | | | | | |
| 7 | | | | | |
| 8 | | | | | |
| 9 | | | | | |
| 10 | | | | | |
| 11 | | | | | |
| 12 | | | | | |
| 13 | | | | | |
| 14 | | | | | |
| 15 | | | | | |
| 16 | | | | | |
| 17 | | | | | |
| 18 | | | | | |
| 19 | | | | | |
| 20 | | | | | |
| 21 | | | | | |
| 22 | | | | | |
| 23 | | | | | |
| 24 | | | | | |
| 25 | | | | | |
| 26 | | | | | |
| 27 | | | | | |
| 28 | | | | | |
| 29 | | | | | |
| 30 | | | | | |
| 31 | | | | | |
| 32 | | | | | |
| 33 | | | | | |
| 34 | | | | | |
| 35 | | | | | |
| 36 | | | | | |
| 37 | | | | | |
| 38 | | | | | |
| 39 | | | | | |
| 40 | | | | | |
| 41 | | | | | |
| 42 | | | | | |
| 43 | | | | | |
| 44 | | | | | |
| 45 | | | | | |
| 46 | | | | | |
| 47 | | | | | |
| 48 | | | | | |
| 49 | | | | | |
| 50 | | | | | |
3
‘Hits’ in red are the additional ‘hits’ identified without replicate exclusion.

## Slide 4
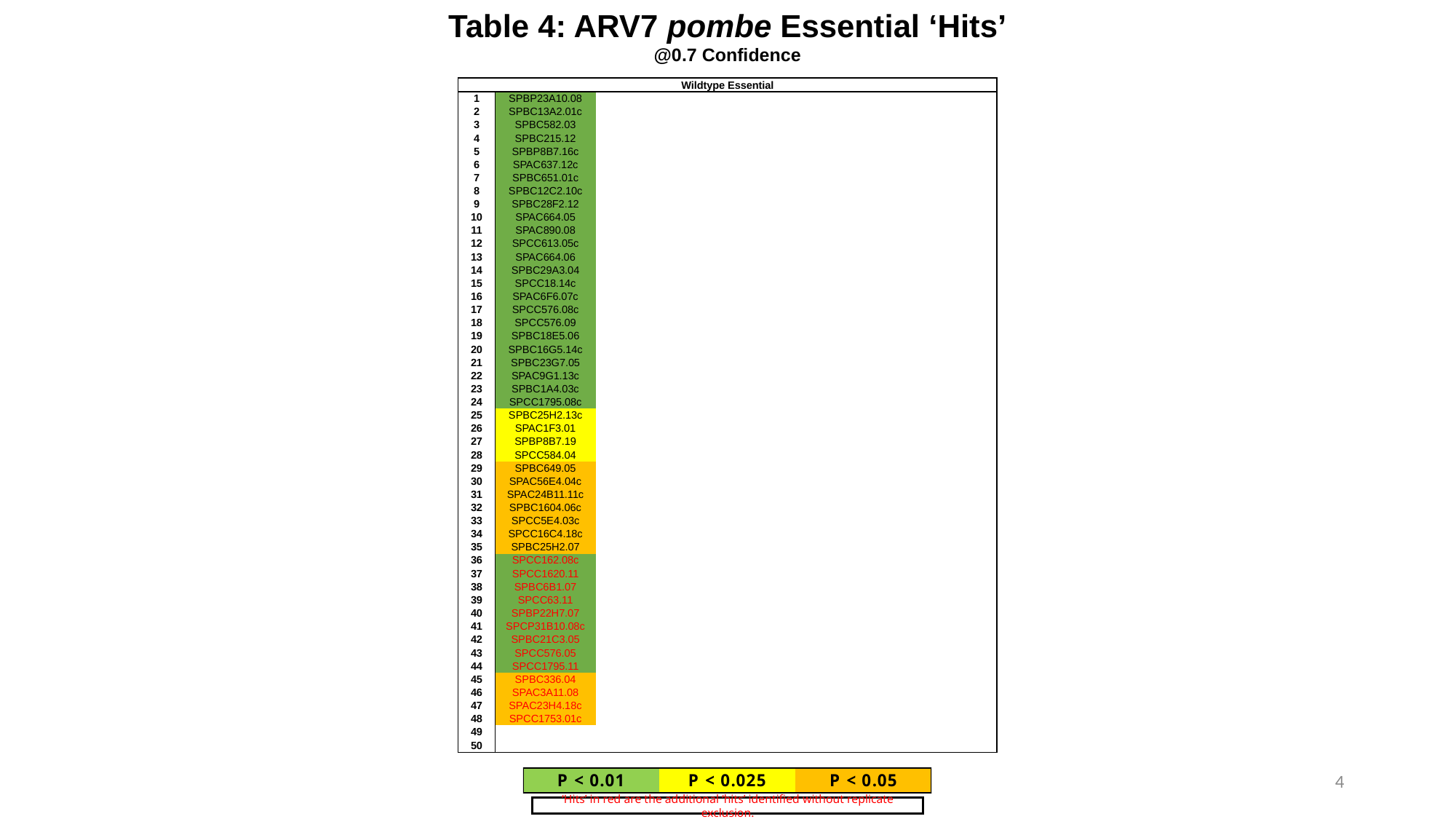

Table 4: ARV7 pombe Essential ‘Hits’
@0.7 Confidence
| Wildtype Essential | | | | | |
| --- | --- | --- | --- | --- | --- |
| 1 | SPBP23A10.08 | | | | |
| 2 | SPBC13A2.01c | | | | |
| 3 | SPBC582.03 | | | | |
| 4 | SPBC215.12 | | | | |
| 5 | SPBP8B7.16c | | | | |
| 6 | SPAC637.12c | | | | |
| 7 | SPBC651.01c | | | | |
| 8 | SPBC12C2.10c | | | | |
| 9 | SPBC28F2.12 | | | | |
| 10 | SPAC664.05 | | | | |
| 11 | SPAC890.08 | | | | |
| 12 | SPCC613.05c | | | | |
| 13 | SPAC664.06 | | | | |
| 14 | SPBC29A3.04 | | | | |
| 15 | SPCC18.14c | | | | |
| 16 | SPAC6F6.07c | | | | |
| 17 | SPCC576.08c | | | | |
| 18 | SPCC576.09 | | | | |
| 19 | SPBC18E5.06 | | | | |
| 20 | SPBC16G5.14c | | | | |
| 21 | SPBC23G7.05 | | | | |
| 22 | SPAC9G1.13c | | | | |
| 23 | SPBC1A4.03c | | | | |
| 24 | SPCC1795.08c | | | | |
| 25 | SPBC25H2.13c | | | | |
| 26 | SPAC1F3.01 | | | | |
| 27 | SPBP8B7.19 | | | | |
| 28 | SPCC584.04 | | | | |
| 29 | SPBC649.05 | | | | |
| 30 | SPAC56E4.04c | | | | |
| 31 | SPAC24B11.11c | | | | |
| 32 | SPBC1604.06c | | | | |
| 33 | SPCC5E4.03c | | | | |
| 34 | SPCC16C4.18c | | | | |
| 35 | SPBC25H2.07 | | | | |
| 36 | SPCC162.08c | | | | |
| 37 | SPCC1620.11 | | | | |
| 38 | SPBC6B1.07 | | | | |
| 39 | SPCC63.11 | | | | |
| 40 | SPBP22H7.07 | | | | |
| 41 | SPCP31B10.08c | | | | |
| 42 | SPBC21C3.05 | | | | |
| 43 | SPCC576.05 | | | | |
| 44 | SPCC1795.11 | | | | |
| 45 | SPBC336.04 | | | | |
| 46 | SPAC3A11.08 | | | | |
| 47 | SPAC23H4.18c | | | | |
| 48 | SPCC1753.01c | | | | |
| 49 | | | | | |
| 50 | | | | | |
4
‘Hits’ in red are the additional ‘hits’ identified without replicate exclusion.

## Slide 5
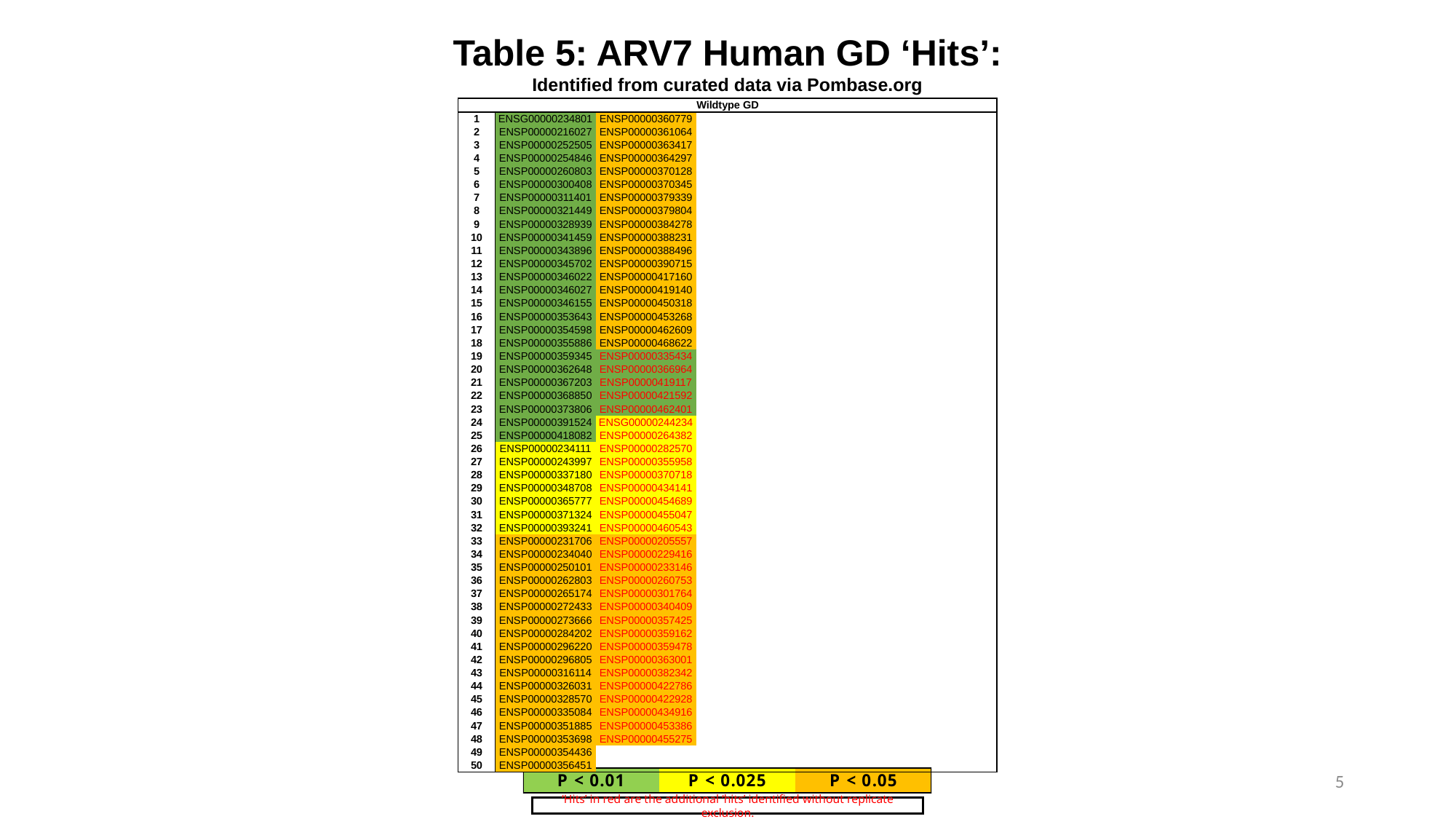

Table 5: ARV7 Human GD ‘Hits’:
Identified from curated data via Pombase.org
| Wildtype GD | | | | | |
| --- | --- | --- | --- | --- | --- |
| 1 | ENSG00000234801 | ENSP00000360779 | | | |
| 2 | ENSP00000216027 | ENSP00000361064 | | | |
| 3 | ENSP00000252505 | ENSP00000363417 | | | |
| 4 | ENSP00000254846 | ENSP00000364297 | | | |
| 5 | ENSP00000260803 | ENSP00000370128 | | | |
| 6 | ENSP00000300408 | ENSP00000370345 | | | |
| 7 | ENSP00000311401 | ENSP00000379339 | | | |
| 8 | ENSP00000321449 | ENSP00000379804 | | | |
| 9 | ENSP00000328939 | ENSP00000384278 | | | |
| 10 | ENSP00000341459 | ENSP00000388231 | | | |
| 11 | ENSP00000343896 | ENSP00000388496 | | | |
| 12 | ENSP00000345702 | ENSP00000390715 | | | |
| 13 | ENSP00000346022 | ENSP00000417160 | | | |
| 14 | ENSP00000346027 | ENSP00000419140 | | | |
| 15 | ENSP00000346155 | ENSP00000450318 | | | |
| 16 | ENSP00000353643 | ENSP00000453268 | | | |
| 17 | ENSP00000354598 | ENSP00000462609 | | | |
| 18 | ENSP00000355886 | ENSP00000468622 | | | |
| 19 | ENSP00000359345 | ENSP00000335434 | | | |
| 20 | ENSP00000362648 | ENSP00000366964 | | | |
| 21 | ENSP00000367203 | ENSP00000419117 | | | |
| 22 | ENSP00000368850 | ENSP00000421592 | | | |
| 23 | ENSP00000373806 | ENSP00000462401 | | | |
| 24 | ENSP00000391524 | ENSG00000244234 | | | |
| 25 | ENSP00000418082 | ENSP00000264382 | | | |
| 26 | ENSP00000234111 | ENSP00000282570 | | | |
| 27 | ENSP00000243997 | ENSP00000355958 | | | |
| 28 | ENSP00000337180 | ENSP00000370718 | | | |
| 29 | ENSP00000348708 | ENSP00000434141 | | | |
| 30 | ENSP00000365777 | ENSP00000454689 | | | |
| 31 | ENSP00000371324 | ENSP00000455047 | | | |
| 32 | ENSP00000393241 | ENSP00000460543 | | | |
| 33 | ENSP00000231706 | ENSP00000205557 | | | |
| 34 | ENSP00000234040 | ENSP00000229416 | | | |
| 35 | ENSP00000250101 | ENSP00000233146 | | | |
| 36 | ENSP00000262803 | ENSP00000260753 | | | |
| 37 | ENSP00000265174 | ENSP00000301764 | | | |
| 38 | ENSP00000272433 | ENSP00000340409 | | | |
| 39 | ENSP00000273666 | ENSP00000357425 | | | |
| 40 | ENSP00000284202 | ENSP00000359162 | | | |
| 41 | ENSP00000296220 | ENSP00000359478 | | | |
| 42 | ENSP00000296805 | ENSP00000363001 | | | |
| 43 | ENSP00000316114 | ENSP00000382342 | | | |
| 44 | ENSP00000326031 | ENSP00000422786 | | | |
| 45 | ENSP00000328570 | ENSP00000422928 | | | |
| 46 | ENSP00000335084 | ENSP00000434916 | | | |
| 47 | ENSP00000351885 | ENSP00000453386 | | | |
| 48 | ENSP00000353698 | ENSP00000455275 | | | |
| 49 | ENSP00000354436 | | | | |
| 50 | ENSP00000356451 | | | | |
5
‘Hits’ in red are the additional ‘hits’ identified without replicate exclusion.

## Slide 6
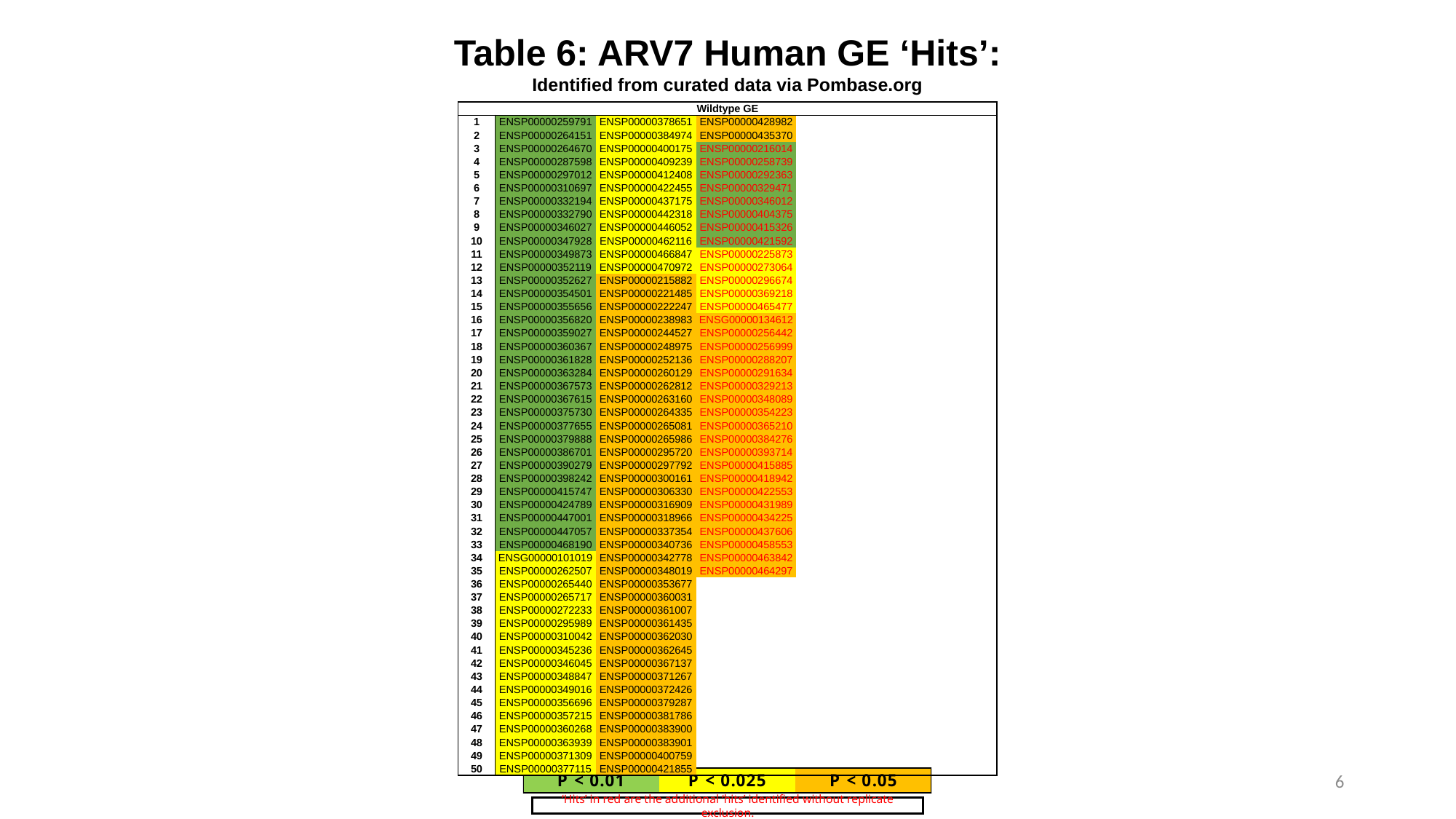

Table 6: ARV7 Human GE ‘Hits’:
Identified from curated data via Pombase.org
| Wildtype GE | | | | | |
| --- | --- | --- | --- | --- | --- |
| 1 | ENSP00000259791 | ENSP00000378651 | ENSP00000428982 | | |
| 2 | ENSP00000264151 | ENSP00000384974 | ENSP00000435370 | | |
| 3 | ENSP00000264670 | ENSP00000400175 | ENSP00000216014 | | |
| 4 | ENSP00000287598 | ENSP00000409239 | ENSP00000258739 | | |
| 5 | ENSP00000297012 | ENSP00000412408 | ENSP00000292363 | | |
| 6 | ENSP00000310697 | ENSP00000422455 | ENSP00000329471 | | |
| 7 | ENSP00000332194 | ENSP00000437175 | ENSP00000346012 | | |
| 8 | ENSP00000332790 | ENSP00000442318 | ENSP00000404375 | | |
| 9 | ENSP00000346027 | ENSP00000446052 | ENSP00000415326 | | |
| 10 | ENSP00000347928 | ENSP00000462116 | ENSP00000421592 | | |
| 11 | ENSP00000349873 | ENSP00000466847 | ENSP00000225873 | | |
| 12 | ENSP00000352119 | ENSP00000470972 | ENSP00000273064 | | |
| 13 | ENSP00000352627 | ENSP00000215882 | ENSP00000296674 | | |
| 14 | ENSP00000354501 | ENSP00000221485 | ENSP00000369218 | | |
| 15 | ENSP00000355656 | ENSP00000222247 | ENSP00000465477 | | |
| 16 | ENSP00000356820 | ENSP00000238983 | ENSG00000134612 | | |
| 17 | ENSP00000359027 | ENSP00000244527 | ENSP00000256442 | | |
| 18 | ENSP00000360367 | ENSP00000248975 | ENSP00000256999 | | |
| 19 | ENSP00000361828 | ENSP00000252136 | ENSP00000288207 | | |
| 20 | ENSP00000363284 | ENSP00000260129 | ENSP00000291634 | | |
| 21 | ENSP00000367573 | ENSP00000262812 | ENSP00000329213 | | |
| 22 | ENSP00000367615 | ENSP00000263160 | ENSP00000348089 | | |
| 23 | ENSP00000375730 | ENSP00000264335 | ENSP00000354223 | | |
| 24 | ENSP00000377655 | ENSP00000265081 | ENSP00000365210 | | |
| 25 | ENSP00000379888 | ENSP00000265986 | ENSP00000384276 | | |
| 26 | ENSP00000386701 | ENSP00000295720 | ENSP00000393714 | | |
| 27 | ENSP00000390279 | ENSP00000297792 | ENSP00000415885 | | |
| 28 | ENSP00000398242 | ENSP00000300161 | ENSP00000418942 | | |
| 29 | ENSP00000415747 | ENSP00000306330 | ENSP00000422553 | | |
| 30 | ENSP00000424789 | ENSP00000316909 | ENSP00000431989 | | |
| 31 | ENSP00000447001 | ENSP00000318966 | ENSP00000434225 | | |
| 32 | ENSP00000447057 | ENSP00000337354 | ENSP00000437606 | | |
| 33 | ENSP00000468190 | ENSP00000340736 | ENSP00000458553 | | |
| 34 | ENSG00000101019 | ENSP00000342778 | ENSP00000463842 | | |
| 35 | ENSP00000262507 | ENSP00000348019 | ENSP00000464297 | | |
| 36 | ENSP00000265440 | ENSP00000353677 | | | |
| 37 | ENSP00000265717 | ENSP00000360031 | | | |
| 38 | ENSP00000272233 | ENSP00000361007 | | | |
| 39 | ENSP00000295989 | ENSP00000361435 | | | |
| 40 | ENSP00000310042 | ENSP00000362030 | | | |
| 41 | ENSP00000345236 | ENSP00000362645 | | | |
| 42 | ENSP00000346045 | ENSP00000367137 | | | |
| 43 | ENSP00000348847 | ENSP00000371267 | | | |
| 44 | ENSP00000349016 | ENSP00000372426 | | | |
| 45 | ENSP00000356696 | ENSP00000379287 | | | |
| 46 | ENSP00000357215 | ENSP00000381786 | | | |
| 47 | ENSP00000360268 | ENSP00000383900 | | | |
| 48 | ENSP00000363939 | ENSP00000383901 | | | |
| 49 | ENSP00000371309 | ENSP00000400759 | | | |
| 50 | ENSP00000377115 | ENSP00000421855 | | | |
6
‘Hits’ in red are the additional ‘hits’ identified without replicate exclusion.

## Slide 7
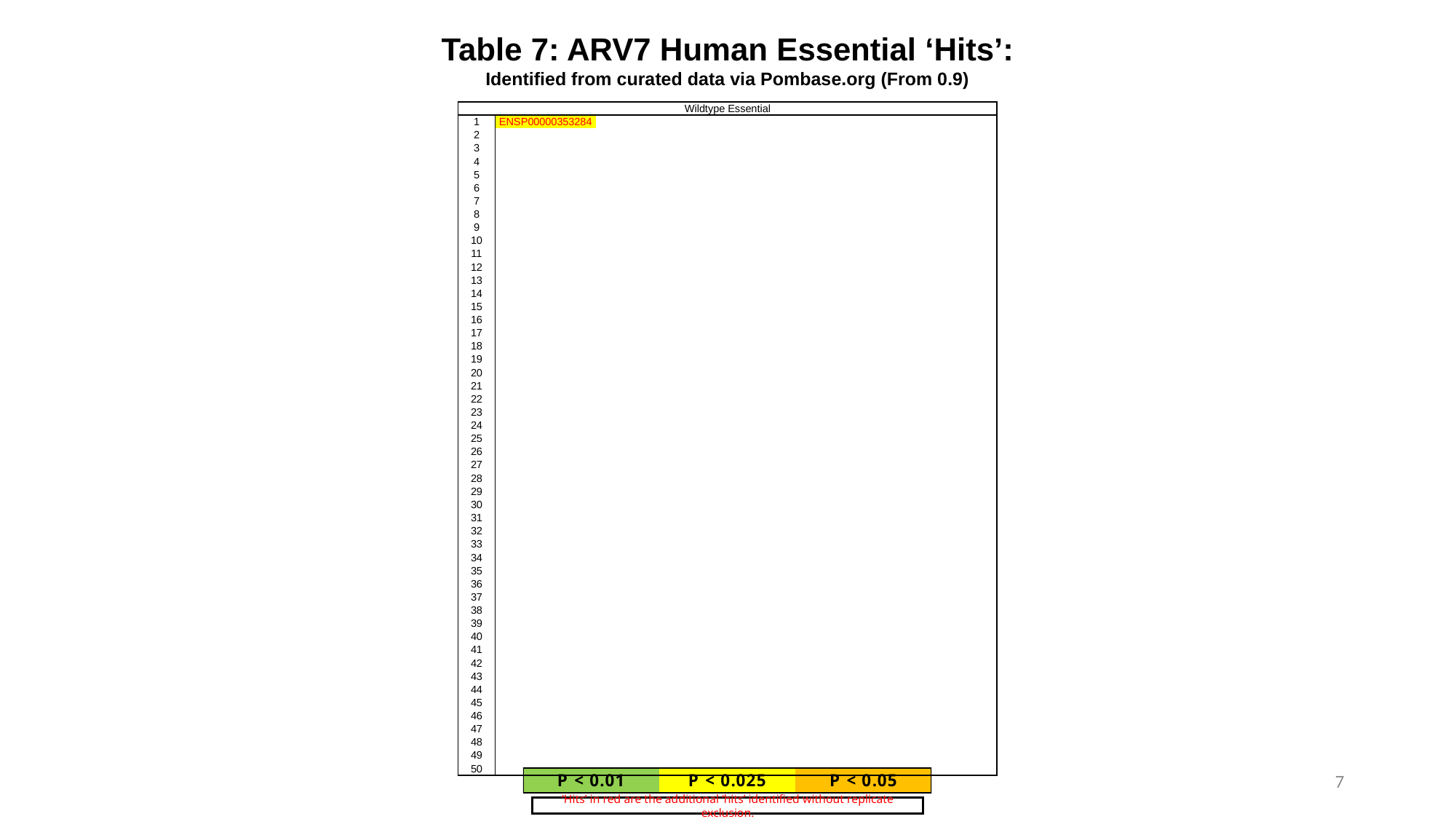

Table 7: ARV7 Human Essential ‘Hits’:
Identified from curated data via Pombase.org (From 0.9)
| Wildtype Essential | | | | | |
| --- | --- | --- | --- | --- | --- |
| 1 | ENSP00000353284 | | | | |
| 2 | | | | | |
| 3 | | | | | |
| 4 | | | | | |
| 5 | | | | | |
| 6 | | | | | |
| 7 | | | | | |
| 8 | | | | | |
| 9 | | | | | |
| 10 | | | | | |
| 11 | | | | | |
| 12 | | | | | |
| 13 | | | | | |
| 14 | | | | | |
| 15 | | | | | |
| 16 | | | | | |
| 17 | | | | | |
| 18 | | | | | |
| 19 | | | | | |
| 20 | | | | | |
| 21 | | | | | |
| 22 | | | | | |
| 23 | | | | | |
| 24 | | | | | |
| 25 | | | | | |
| 26 | | | | | |
| 27 | | | | | |
| 28 | | | | | |
| 29 | | | | | |
| 30 | | | | | |
| 31 | | | | | |
| 32 | | | | | |
| 33 | | | | | |
| 34 | | | | | |
| 35 | | | | | |
| 36 | | | | | |
| 37 | | | | | |
| 38 | | | | | |
| 39 | | | | | |
| 40 | | | | | |
| 41 | | | | | |
| 42 | | | | | |
| 43 | | | | | |
| 44 | | | | | |
| 45 | | | | | |
| 46 | | | | | |
| 47 | | | | | |
| 48 | | | | | |
| 49 | | | | | |
| 50 | | | | | |
7
‘Hits’ in red are the additional ‘hits’ identified without replicate exclusion.

## Slide 8
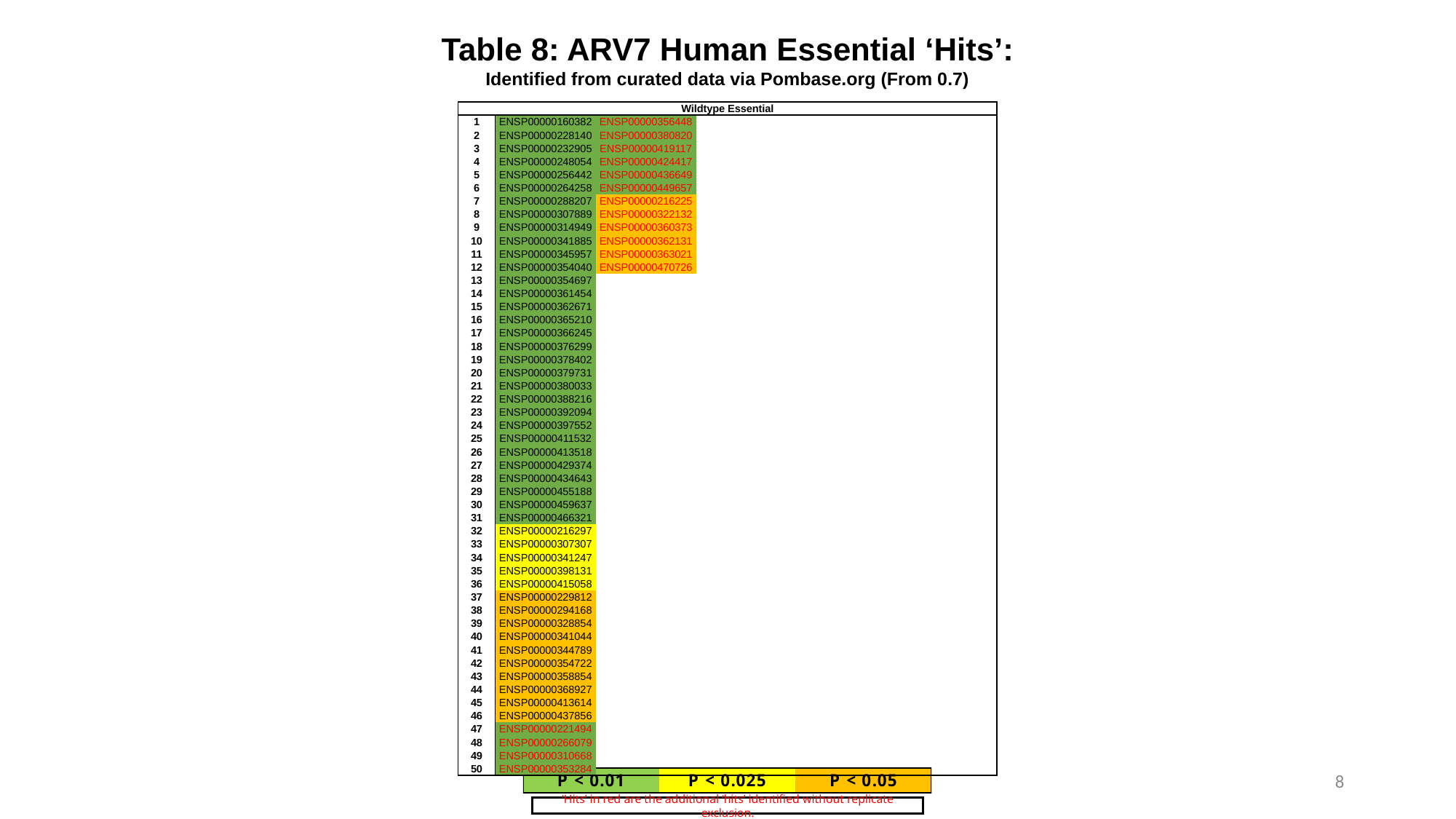

Table 8: ARV7 Human Essential ‘Hits’:
Identified from curated data via Pombase.org (From 0.7)
| Wildtype Essential | | | | | |
| --- | --- | --- | --- | --- | --- |
| 1 | ENSP00000160382 | ENSP00000356448 | | | |
| 2 | ENSP00000228140 | ENSP00000380820 | | | |
| 3 | ENSP00000232905 | ENSP00000419117 | | | |
| 4 | ENSP00000248054 | ENSP00000424417 | | | |
| 5 | ENSP00000256442 | ENSP00000436649 | | | |
| 6 | ENSP00000264258 | ENSP00000449657 | | | |
| 7 | ENSP00000288207 | ENSP00000216225 | | | |
| 8 | ENSP00000307889 | ENSP00000322132 | | | |
| 9 | ENSP00000314949 | ENSP00000360373 | | | |
| 10 | ENSP00000341885 | ENSP00000362131 | | | |
| 11 | ENSP00000345957 | ENSP00000363021 | | | |
| 12 | ENSP00000354040 | ENSP00000470726 | | | |
| 13 | ENSP00000354697 | | | | |
| 14 | ENSP00000361454 | | | | |
| 15 | ENSP00000362671 | | | | |
| 16 | ENSP00000365210 | | | | |
| 17 | ENSP00000366245 | | | | |
| 18 | ENSP00000376299 | | | | |
| 19 | ENSP00000378402 | | | | |
| 20 | ENSP00000379731 | | | | |
| 21 | ENSP00000380033 | | | | |
| 22 | ENSP00000388216 | | | | |
| 23 | ENSP00000392094 | | | | |
| 24 | ENSP00000397552 | | | | |
| 25 | ENSP00000411532 | | | | |
| 26 | ENSP00000413518 | | | | |
| 27 | ENSP00000429374 | | | | |
| 28 | ENSP00000434643 | | | | |
| 29 | ENSP00000455188 | | | | |
| 30 | ENSP00000459637 | | | | |
| 31 | ENSP00000466321 | | | | |
| 32 | ENSP00000216297 | | | | |
| 33 | ENSP00000307307 | | | | |
| 34 | ENSP00000341247 | | | | |
| 35 | ENSP00000398131 | | | | |
| 36 | ENSP00000415058 | | | | |
| 37 | ENSP00000229812 | | | | |
| 38 | ENSP00000294168 | | | | |
| 39 | ENSP00000328854 | | | | |
| 40 | ENSP00000341044 | | | | |
| 41 | ENSP00000344789 | | | | |
| 42 | ENSP00000354722 | | | | |
| 43 | ENSP00000358854 | | | | |
| 44 | ENSP00000368927 | | | | |
| 45 | ENSP00000413614 | | | | |
| 46 | ENSP00000437856 | | | | |
| 47 | ENSP00000221494 | | | | |
| 48 | ENSP00000266079 | | | | |
| 49 | ENSP00000310668 | | | | |
| 50 | ENSP00000353284 | | | | |
8
‘Hits’ in red are the additional ‘hits’ identified without replicate exclusion.

## Slide 9
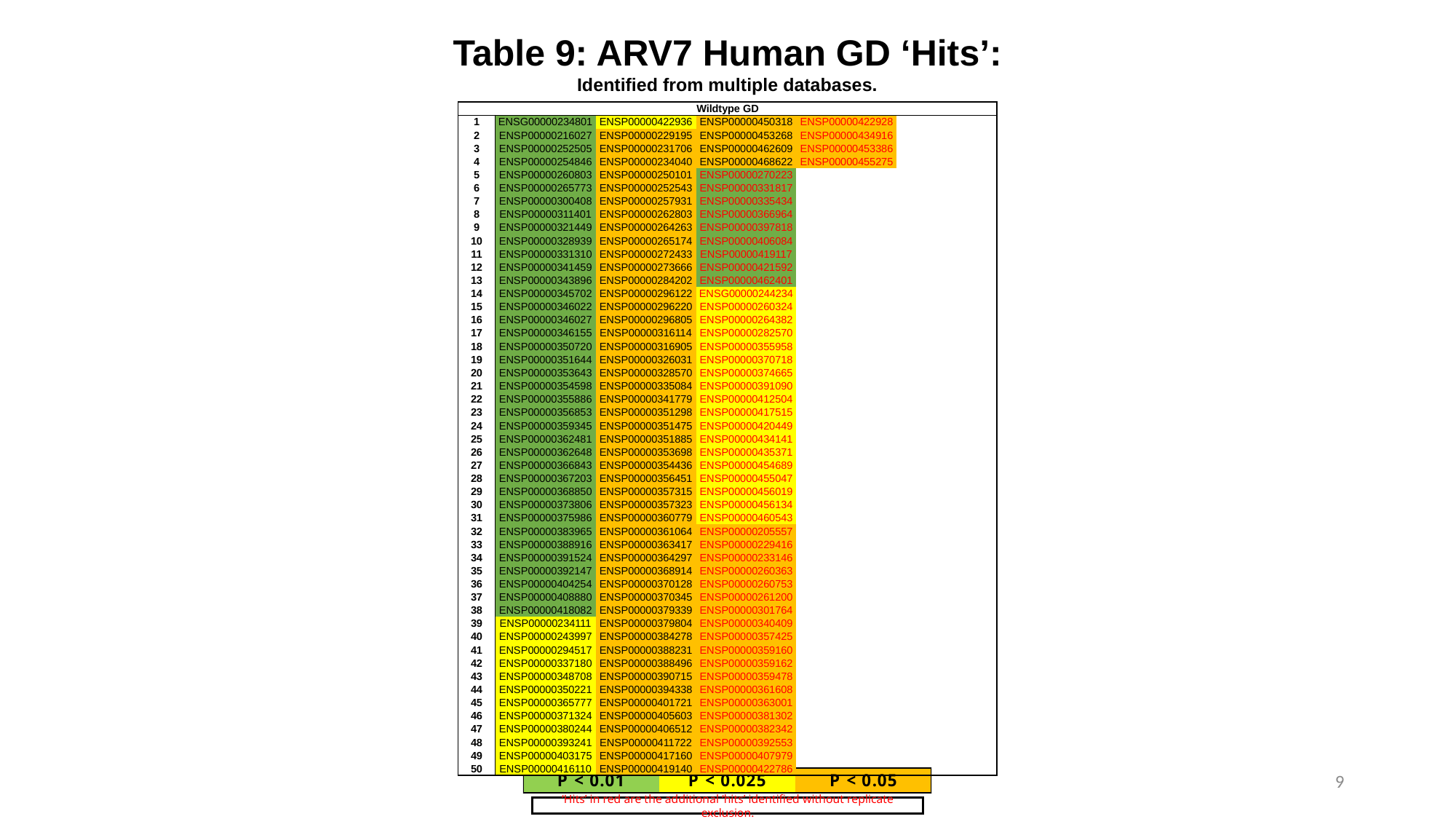

Table 9: ARV7 Human GD ‘Hits’:
Identified from multiple databases.
| Wildtype GD | | | | | |
| --- | --- | --- | --- | --- | --- |
| 1 | ENSG00000234801 | ENSP00000422936 | ENSP00000450318 | ENSP00000422928 | |
| 2 | ENSP00000216027 | ENSP00000229195 | ENSP00000453268 | ENSP00000434916 | |
| 3 | ENSP00000252505 | ENSP00000231706 | ENSP00000462609 | ENSP00000453386 | |
| 4 | ENSP00000254846 | ENSP00000234040 | ENSP00000468622 | ENSP00000455275 | |
| 5 | ENSP00000260803 | ENSP00000250101 | ENSP00000270223 | | |
| 6 | ENSP00000265773 | ENSP00000252543 | ENSP00000331817 | | |
| 7 | ENSP00000300408 | ENSP00000257931 | ENSP00000335434 | | |
| 8 | ENSP00000311401 | ENSP00000262803 | ENSP00000366964 | | |
| 9 | ENSP00000321449 | ENSP00000264263 | ENSP00000397818 | | |
| 10 | ENSP00000328939 | ENSP00000265174 | ENSP00000406084 | | |
| 11 | ENSP00000331310 | ENSP00000272433 | ENSP00000419117 | | |
| 12 | ENSP00000341459 | ENSP00000273666 | ENSP00000421592 | | |
| 13 | ENSP00000343896 | ENSP00000284202 | ENSP00000462401 | | |
| 14 | ENSP00000345702 | ENSP00000296122 | ENSG00000244234 | | |
| 15 | ENSP00000346022 | ENSP00000296220 | ENSP00000260324 | | |
| 16 | ENSP00000346027 | ENSP00000296805 | ENSP00000264382 | | |
| 17 | ENSP00000346155 | ENSP00000316114 | ENSP00000282570 | | |
| 18 | ENSP00000350720 | ENSP00000316905 | ENSP00000355958 | | |
| 19 | ENSP00000351644 | ENSP00000326031 | ENSP00000370718 | | |
| 20 | ENSP00000353643 | ENSP00000328570 | ENSP00000374665 | | |
| 21 | ENSP00000354598 | ENSP00000335084 | ENSP00000391090 | | |
| 22 | ENSP00000355886 | ENSP00000341779 | ENSP00000412504 | | |
| 23 | ENSP00000356853 | ENSP00000351298 | ENSP00000417515 | | |
| 24 | ENSP00000359345 | ENSP00000351475 | ENSP00000420449 | | |
| 25 | ENSP00000362481 | ENSP00000351885 | ENSP00000434141 | | |
| 26 | ENSP00000362648 | ENSP00000353698 | ENSP00000435371 | | |
| 27 | ENSP00000366843 | ENSP00000354436 | ENSP00000454689 | | |
| 28 | ENSP00000367203 | ENSP00000356451 | ENSP00000455047 | | |
| 29 | ENSP00000368850 | ENSP00000357315 | ENSP00000456019 | | |
| 30 | ENSP00000373806 | ENSP00000357323 | ENSP00000456134 | | |
| 31 | ENSP00000375986 | ENSP00000360779 | ENSP00000460543 | | |
| 32 | ENSP00000383965 | ENSP00000361064 | ENSP00000205557 | | |
| 33 | ENSP00000388916 | ENSP00000363417 | ENSP00000229416 | | |
| 34 | ENSP00000391524 | ENSP00000364297 | ENSP00000233146 | | |
| 35 | ENSP00000392147 | ENSP00000368914 | ENSP00000260363 | | |
| 36 | ENSP00000404254 | ENSP00000370128 | ENSP00000260753 | | |
| 37 | ENSP00000408880 | ENSP00000370345 | ENSP00000261200 | | |
| 38 | ENSP00000418082 | ENSP00000379339 | ENSP00000301764 | | |
| 39 | ENSP00000234111 | ENSP00000379804 | ENSP00000340409 | | |
| 40 | ENSP00000243997 | ENSP00000384278 | ENSP00000357425 | | |
| 41 | ENSP00000294517 | ENSP00000388231 | ENSP00000359160 | | |
| 42 | ENSP00000337180 | ENSP00000388496 | ENSP00000359162 | | |
| 43 | ENSP00000348708 | ENSP00000390715 | ENSP00000359478 | | |
| 44 | ENSP00000350221 | ENSP00000394338 | ENSP00000361608 | | |
| 45 | ENSP00000365777 | ENSP00000401721 | ENSP00000363001 | | |
| 46 | ENSP00000371324 | ENSP00000405603 | ENSP00000381302 | | |
| 47 | ENSP00000380244 | ENSP00000406512 | ENSP00000382342 | | |
| 48 | ENSP00000393241 | ENSP00000411722 | ENSP00000392553 | | |
| 49 | ENSP00000403175 | ENSP00000417160 | ENSP00000407979 | | |
| 50 | ENSP00000416110 | ENSP00000419140 | ENSP00000422786 | | |
9
‘Hits’ in red are the additional ‘hits’ identified without replicate exclusion.

## Slide 10
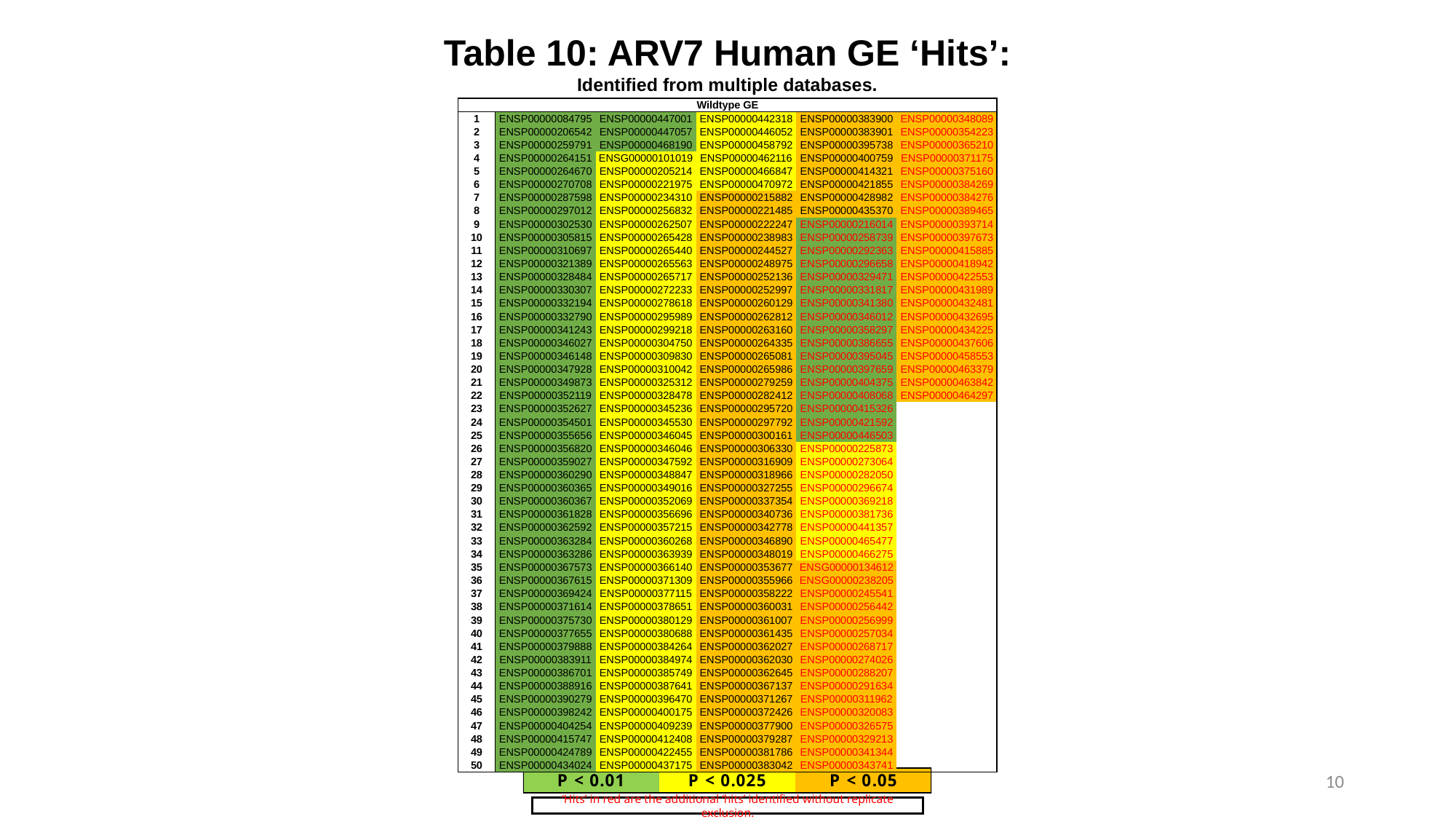

Table 10: ARV7 Human GE ‘Hits’:
Identified from multiple databases.
| Wildtype GE | | | | | |
| --- | --- | --- | --- | --- | --- |
| 1 | ENSP00000084795 | ENSP00000447001 | ENSP00000442318 | ENSP00000383900 | ENSP00000348089 |
| 2 | ENSP00000206542 | ENSP00000447057 | ENSP00000446052 | ENSP00000383901 | ENSP00000354223 |
| 3 | ENSP00000259791 | ENSP00000468190 | ENSP00000458792 | ENSP00000395738 | ENSP00000365210 |
| 4 | ENSP00000264151 | ENSG00000101019 | ENSP00000462116 | ENSP00000400759 | ENSP00000371175 |
| 5 | ENSP00000264670 | ENSP00000205214 | ENSP00000466847 | ENSP00000414321 | ENSP00000375160 |
| 6 | ENSP00000270708 | ENSP00000221975 | ENSP00000470972 | ENSP00000421855 | ENSP00000384269 |
| 7 | ENSP00000287598 | ENSP00000234310 | ENSP00000215882 | ENSP00000428982 | ENSP00000384276 |
| 8 | ENSP00000297012 | ENSP00000256832 | ENSP00000221485 | ENSP00000435370 | ENSP00000389465 |
| 9 | ENSP00000302530 | ENSP00000262507 | ENSP00000222247 | ENSP00000216014 | ENSP00000393714 |
| 10 | ENSP00000305815 | ENSP00000265428 | ENSP00000238983 | ENSP00000258739 | ENSP00000397673 |
| 11 | ENSP00000310697 | ENSP00000265440 | ENSP00000244527 | ENSP00000292363 | ENSP00000415885 |
| 12 | ENSP00000321389 | ENSP00000265563 | ENSP00000248975 | ENSP00000296658 | ENSP00000418942 |
| 13 | ENSP00000328484 | ENSP00000265717 | ENSP00000252136 | ENSP00000329471 | ENSP00000422553 |
| 14 | ENSP00000330307 | ENSP00000272233 | ENSP00000252997 | ENSP00000331817 | ENSP00000431989 |
| 15 | ENSP00000332194 | ENSP00000278618 | ENSP00000260129 | ENSP00000341380 | ENSP00000432481 |
| 16 | ENSP00000332790 | ENSP00000295989 | ENSP00000262812 | ENSP00000346012 | ENSP00000432695 |
| 17 | ENSP00000341243 | ENSP00000299218 | ENSP00000263160 | ENSP00000358297 | ENSP00000434225 |
| 18 | ENSP00000346027 | ENSP00000304750 | ENSP00000264335 | ENSP00000386655 | ENSP00000437606 |
| 19 | ENSP00000346148 | ENSP00000309830 | ENSP00000265081 | ENSP00000395045 | ENSP00000458553 |
| 20 | ENSP00000347928 | ENSP00000310042 | ENSP00000265986 | ENSP00000397659 | ENSP00000463379 |
| 21 | ENSP00000349873 | ENSP00000325312 | ENSP00000279259 | ENSP00000404375 | ENSP00000463842 |
| 22 | ENSP00000352119 | ENSP00000328478 | ENSP00000282412 | ENSP00000408068 | ENSP00000464297 |
| 23 | ENSP00000352627 | ENSP00000345236 | ENSP00000295720 | ENSP00000415326 | |
| 24 | ENSP00000354501 | ENSP00000345530 | ENSP00000297792 | ENSP00000421592 | |
| 25 | ENSP00000355656 | ENSP00000346045 | ENSP00000300161 | ENSP00000446503 | |
| 26 | ENSP00000356820 | ENSP00000346046 | ENSP00000306330 | ENSP00000225873 | |
| 27 | ENSP00000359027 | ENSP00000347592 | ENSP00000316909 | ENSP00000273064 | |
| 28 | ENSP00000360290 | ENSP00000348847 | ENSP00000318966 | ENSP00000282050 | |
| 29 | ENSP00000360365 | ENSP00000349016 | ENSP00000327255 | ENSP00000296674 | |
| 30 | ENSP00000360367 | ENSP00000352069 | ENSP00000337354 | ENSP00000369218 | |
| 31 | ENSP00000361828 | ENSP00000356696 | ENSP00000340736 | ENSP00000381736 | |
| 32 | ENSP00000362592 | ENSP00000357215 | ENSP00000342778 | ENSP00000441357 | |
| 33 | ENSP00000363284 | ENSP00000360268 | ENSP00000346890 | ENSP00000465477 | |
| 34 | ENSP00000363286 | ENSP00000363939 | ENSP00000348019 | ENSP00000466275 | |
| 35 | ENSP00000367573 | ENSP00000366140 | ENSP00000353677 | ENSG00000134612 | |
| 36 | ENSP00000367615 | ENSP00000371309 | ENSP00000355966 | ENSG00000238205 | |
| 37 | ENSP00000369424 | ENSP00000377115 | ENSP00000358222 | ENSP00000245541 | |
| 38 | ENSP00000371614 | ENSP00000378651 | ENSP00000360031 | ENSP00000256442 | |
| 39 | ENSP00000375730 | ENSP00000380129 | ENSP00000361007 | ENSP00000256999 | |
| 40 | ENSP00000377655 | ENSP00000380688 | ENSP00000361435 | ENSP00000257034 | |
| 41 | ENSP00000379888 | ENSP00000384264 | ENSP00000362027 | ENSP00000268717 | |
| 42 | ENSP00000383911 | ENSP00000384974 | ENSP00000362030 | ENSP00000274026 | |
| 43 | ENSP00000386701 | ENSP00000385749 | ENSP00000362645 | ENSP00000288207 | |
| 44 | ENSP00000388916 | ENSP00000387641 | ENSP00000367137 | ENSP00000291634 | |
| 45 | ENSP00000390279 | ENSP00000396470 | ENSP00000371267 | ENSP00000311962 | |
| 46 | ENSP00000398242 | ENSP00000400175 | ENSP00000372426 | ENSP00000320083 | |
| 47 | ENSP00000404254 | ENSP00000409239 | ENSP00000377900 | ENSP00000326575 | |
| 48 | ENSP00000415747 | ENSP00000412408 | ENSP00000379287 | ENSP00000329213 | |
| 49 | ENSP00000424789 | ENSP00000422455 | ENSP00000381786 | ENSP00000341344 | |
| 50 | ENSP00000434024 | ENSP00000437175 | ENSP00000383042 | ENSP00000343741 | |
10
‘Hits’ in red are the additional ‘hits’ identified without replicate exclusion.

## Slide 11
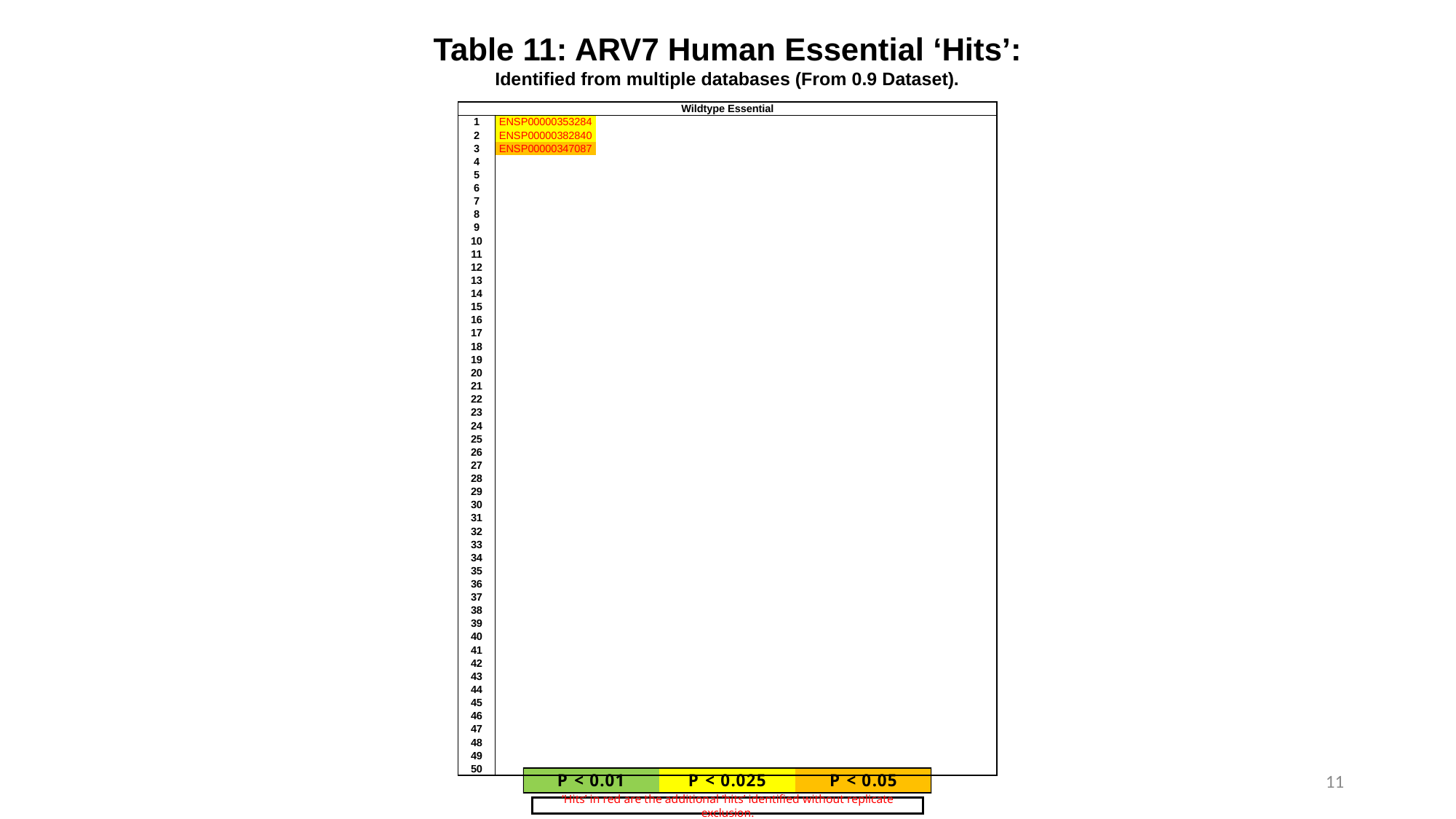

Table 11: ARV7 Human Essential ‘Hits’:
Identified from multiple databases (From 0.9 Dataset).
| Wildtype Essential | | | | | |
| --- | --- | --- | --- | --- | --- |
| 1 | ENSP00000353284 | | | | |
| 2 | ENSP00000382840 | | | | |
| 3 | ENSP00000347087 | | | | |
| 4 | | | | | |
| 5 | | | | | |
| 6 | | | | | |
| 7 | | | | | |
| 8 | | | | | |
| 9 | | | | | |
| 10 | | | | | |
| 11 | | | | | |
| 12 | | | | | |
| 13 | | | | | |
| 14 | | | | | |
| 15 | | | | | |
| 16 | | | | | |
| 17 | | | | | |
| 18 | | | | | |
| 19 | | | | | |
| 20 | | | | | |
| 21 | | | | | |
| 22 | | | | | |
| 23 | | | | | |
| 24 | | | | | |
| 25 | | | | | |
| 26 | | | | | |
| 27 | | | | | |
| 28 | | | | | |
| 29 | | | | | |
| 30 | | | | | |
| 31 | | | | | |
| 32 | | | | | |
| 33 | | | | | |
| 34 | | | | | |
| 35 | | | | | |
| 36 | | | | | |
| 37 | | | | | |
| 38 | | | | | |
| 39 | | | | | |
| 40 | | | | | |
| 41 | | | | | |
| 42 | | | | | |
| 43 | | | | | |
| 44 | | | | | |
| 45 | | | | | |
| 46 | | | | | |
| 47 | | | | | |
| 48 | | | | | |
| 49 | | | | | |
| 50 | | | | | |
11
‘Hits’ in red are the additional ‘hits’ identified without replicate exclusion.

## Slide 12
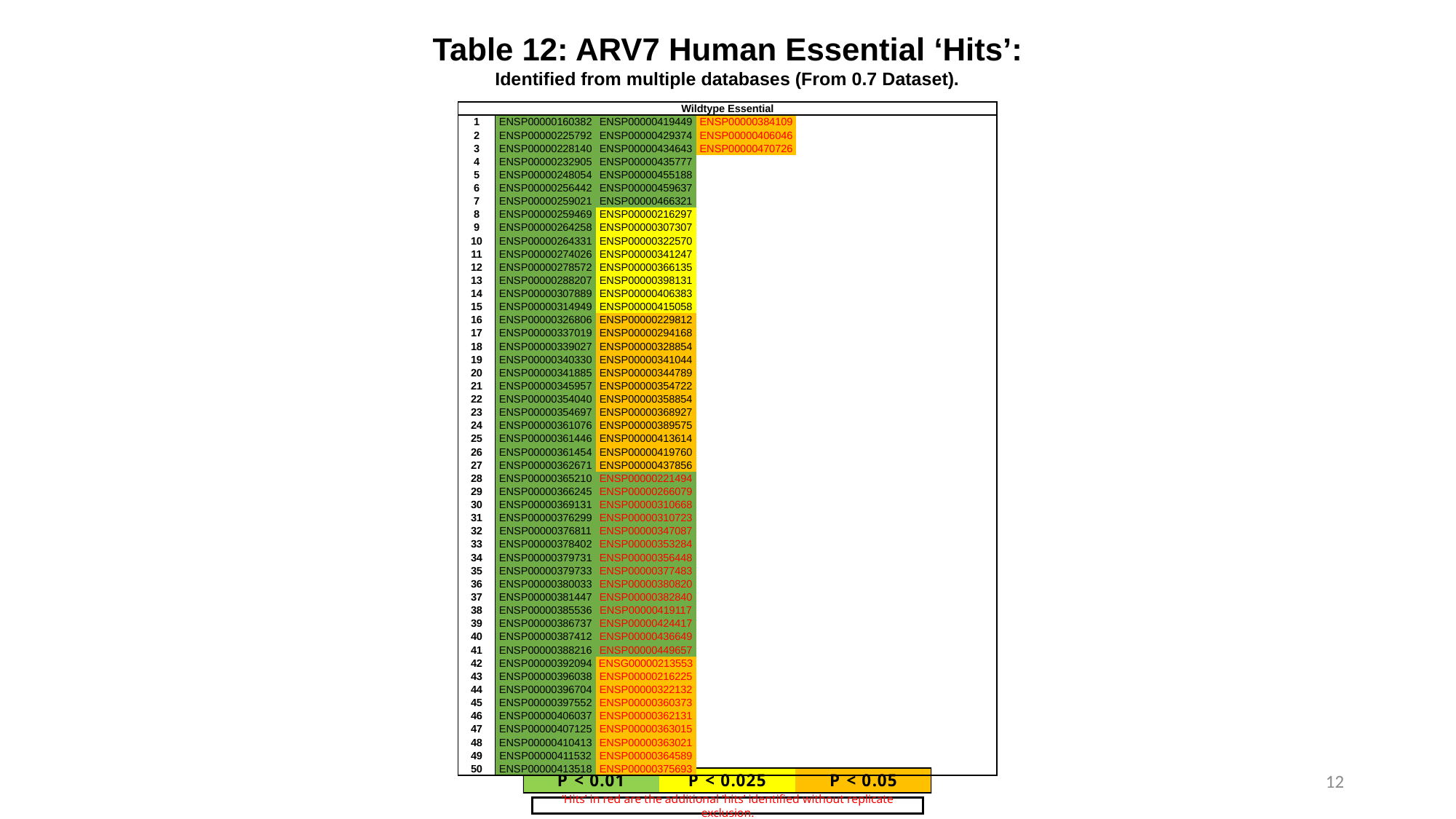

Table 12: ARV7 Human Essential ‘Hits’:
Identified from multiple databases (From 0.7 Dataset).
| Wildtype Essential | | | | | |
| --- | --- | --- | --- | --- | --- |
| 1 | ENSP00000160382 | ENSP00000419449 | ENSP00000384109 | | |
| 2 | ENSP00000225792 | ENSP00000429374 | ENSP00000406046 | | |
| 3 | ENSP00000228140 | ENSP00000434643 | ENSP00000470726 | | |
| 4 | ENSP00000232905 | ENSP00000435777 | | | |
| 5 | ENSP00000248054 | ENSP00000455188 | | | |
| 6 | ENSP00000256442 | ENSP00000459637 | | | |
| 7 | ENSP00000259021 | ENSP00000466321 | | | |
| 8 | ENSP00000259469 | ENSP00000216297 | | | |
| 9 | ENSP00000264258 | ENSP00000307307 | | | |
| 10 | ENSP00000264331 | ENSP00000322570 | | | |
| 11 | ENSP00000274026 | ENSP00000341247 | | | |
| 12 | ENSP00000278572 | ENSP00000366135 | | | |
| 13 | ENSP00000288207 | ENSP00000398131 | | | |
| 14 | ENSP00000307889 | ENSP00000406383 | | | |
| 15 | ENSP00000314949 | ENSP00000415058 | | | |
| 16 | ENSP00000326806 | ENSP00000229812 | | | |
| 17 | ENSP00000337019 | ENSP00000294168 | | | |
| 18 | ENSP00000339027 | ENSP00000328854 | | | |
| 19 | ENSP00000340330 | ENSP00000341044 | | | |
| 20 | ENSP00000341885 | ENSP00000344789 | | | |
| 21 | ENSP00000345957 | ENSP00000354722 | | | |
| 22 | ENSP00000354040 | ENSP00000358854 | | | |
| 23 | ENSP00000354697 | ENSP00000368927 | | | |
| 24 | ENSP00000361076 | ENSP00000389575 | | | |
| 25 | ENSP00000361446 | ENSP00000413614 | | | |
| 26 | ENSP00000361454 | ENSP00000419760 | | | |
| 27 | ENSP00000362671 | ENSP00000437856 | | | |
| 28 | ENSP00000365210 | ENSP00000221494 | | | |
| 29 | ENSP00000366245 | ENSP00000266079 | | | |
| 30 | ENSP00000369131 | ENSP00000310668 | | | |
| 31 | ENSP00000376299 | ENSP00000310723 | | | |
| 32 | ENSP00000376811 | ENSP00000347087 | | | |
| 33 | ENSP00000378402 | ENSP00000353284 | | | |
| 34 | ENSP00000379731 | ENSP00000356448 | | | |
| 35 | ENSP00000379733 | ENSP00000377483 | | | |
| 36 | ENSP00000380033 | ENSP00000380820 | | | |
| 37 | ENSP00000381447 | ENSP00000382840 | | | |
| 38 | ENSP00000385536 | ENSP00000419117 | | | |
| 39 | ENSP00000386737 | ENSP00000424417 | | | |
| 40 | ENSP00000387412 | ENSP00000436649 | | | |
| 41 | ENSP00000388216 | ENSP00000449657 | | | |
| 42 | ENSP00000392094 | ENSG00000213553 | | | |
| 43 | ENSP00000396038 | ENSP00000216225 | | | |
| 44 | ENSP00000396704 | ENSP00000322132 | | | |
| 45 | ENSP00000397552 | ENSP00000360373 | | | |
| 46 | ENSP00000406037 | ENSP00000362131 | | | |
| 47 | ENSP00000407125 | ENSP00000363015 | | | |
| 48 | ENSP00000410413 | ENSP00000363021 | | | |
| 49 | ENSP00000411532 | ENSP00000364589 | | | |
| 50 | ENSP00000413518 | ENSP00000375693 | | | |
12
‘Hits’ in red are the additional ‘hits’ identified without replicate exclusion.
